# Alcoholic extracts of Russian sage ( *Salvia yangii* ) contain bioactive terpenoids with inhibitory activity against grapevine downy mildew ( *Plasmopara viticola* )

**DOI:** 10.1002/ps.70869

**Published:** 2026-05-01

**Authors:** Anna Smaldone, Oscar Giovannini, Martina Magni, Michael Oberhuber, Peter Robatscher, Michele Perazzolli

**Affiliations:** ^1^ Center for Agriculture Food Environment (C3A) University of Trento San Michele all'Adige Italy; ^2^ Laboratory for Flavours and Metabolites, Laimburg Research Centre, Laimburg 6, Pfatten (Vadena) Auer (Ora) Italy; ^3^ Research and Innovation Centre, Fondazione Edmund Mach San Michele all'Adige Italy

**Keywords:** antifungal, bioactive compounds, bioguided separation, plant extracts, *Plasmopara viticola*, *Salvia yangii*, untargeted metabolomics

## Abstract

**BACKGROUND:**

Plant extracts are promising eco‐friendly alternatives to synthetic fungicides for developing sustainable plant protection strategies. Grapevine downy mildew, caused by *Plasmopara viticola*, is a devastating disease that requires frequent fungicide applications, making it an ideal target for plant‐based products. *Salvia officinalis* extracts are known to reduce downy mildew severity, but little is known about the efficacy and chemical composition of other fast‐growing *Salvia* species with low economic value. This study aimed to evaluate the efficacy of alcoholic extracts of Russian sage (*Salvia yangii*) against grapevine downy mildew and to annotate bioactive compounds against *P. viticola*.

**RESULTS:**

Alcoholic extracts from flowers, leaves, and shoots of Russian sage decreased downy mildew severity on grapevine leaf disks. In particular, leaf and flower extracts showed efficacy comparable to that of copper‐based fungicides, whereas stem extract was only partially active. Shoot extracts were fractionated by preparative liquid chromatography (LC), and the activity of the resulting fractions was assessed against *P. viticola* using leaf disk assays. Untargeted metabolomic analyses revealed putative terpenoids as the main components of the active fractions, as determined by gas chromatography–mass spectrometry (GC–MS) and ultra‐high‐pressure liquid chromatography‐high resolution mass spectrometry (UHPLC‐HRMS) analyses. Specifically, 7‐methylrosmanol, 12‐O‐methylcarnosic acid, carnosic acid, and carnosol were identified as the main bioactive compounds against *P. viticola*.

**CONCLUSION:**

Russian sage represents a valuable source of bioactive compounds for sustainable plant protection. © 2026 The Author(s). *Pest Management Science* published by John Wiley & Sons Ltd on behalf of Society of Chemical Industry.

## Graphical abstract

Alcoholic extracts from flowers, leaves, and shoots of Russian sage decreased downy mildew severity on grapevine leaf disks. Fractionation followed by metabolomic analysis revealed putative terpenoids as the main components of the active fractions. Specifically, 7‐methylrosmanol, 12‐O‐methylcarnosic acid, carnosic acid, and carnosol were identified as the main bioactive compounds against *P. viticola*.

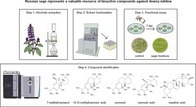

## 1 INTRODUCTION

Crop pathogens and pests reduce the yield and quality of agricultural production, and up to 30% of yield losses were estimated for the five major crops worldwide (i.e., maize, potato, rice, soybean, and wheat).^1^ The application of pesticides is the most successful approach to limit crop losses derived from biotic stresses, with fungicides playing a primary role in managing plant diseases.^2^ The use of conventional plant protection products increased globally by 62% from 2000 to 2021, reaching 3.5 million tons in 2021.^3^ However, the overuse of synthetic chemical fungicides raised concerns about their potential impacts on human health and the environment,^4^ increasing the demand for more sustainable alternatives. The integration of fungicide treatments with the use of bioactive natural products, appropriate cultural practices, disease‐resistant varieties, and biological control agents is required to develop sustainable agricultural systems.^2^ In particular, botanical extracts obtained from medicinal plants (e.g., Apiaceae, Asteraceae, Cupressaceae, Lamiaceae, and Myrtaceae families) were proposed as alternative fungicides against important crop pathogens, such as *Alternaria spp*., *Aspergillus spp*., *Botrytis spp*., *Fusarium spp*., *Phytophthora spp*., *Penicillium spp*., and *Rhizoctonia spp*.^5^ Bioactive compounds of medicinal plants with antifungal activities belong mainly to classes of terpenoids (e.g., polygodial and isodrimeninol from *Drimys winteri* against *Gaeumannomyces graminis*),^6^ phenolic compounds (e.g., dunnione, ferulic acid, gallic acid, and isorhamnetin from *Calceolaria integrifolia* against *Aspergillus spp*., *Fusarium spp*., *Rhizoctonia spp*., and *Trichophyton spp*.),^7^ and alkaloids (e.g., cannin‐6‐one, nigakinone, 4,5‐dimethoxycanthin‐6‐one, 1‐methoxycarbonyl‐β‐carboline, and 1‐methoxycarbonyl‐3‐methoxyl‐β‐carboline, from *Picrasma quassioidesi against Alternaria spp*., *Botrytis cinerea*, *Cochliobolus lunatus*, *Fusarium spp*., *Phytophthora capsici, Verticillium dahlia, and Valsa mali*).^8^ Thus, plant extracts and their purified compounds represent promising alternatives to develop sustainable plant protection products.

Among crop diseases, grapevine downy mildew (caused by *Plasmopara viticola*) is one of the most devastating, and its control is highly dependent on fungicide treatments,^9^ making it an ideal target for exploring plant‐based alternatives for disease control. Grapevine (*Vitis vinifera*) is a major fruit crop worldwide, and most of its cultivars are susceptible to downy mildew.^9^, ^10^ Thus, frequent fungicide applications (e.g., every 7–10 days in seasons and locations with high infection pressure) are required to prevent crop losses due to downy mildew infections.^11^, ^12^ However, several active substances currently used against grapevine downy mildew are under scrutiny for substitution in the European Union (EU), such as fluopicolide, metalaxyl, and copper.^13^ Moreover, copper, which is widely used to control downy mildew in organic viticulture, is currently allowed in the EU up to a maximum of 28 kg ha^−1^ over 7 years,^14^ and further limitations in copper use are expected in the future.^15^ Different plant extracts (e.g., *Equisetum arvense, Frangula alnus, Glycyrrhiza glabra, Inula viscosa, Larix sp., Magnolia officinalis*, *Pinus sp*., *Quillaja saponaria, and Salvia officinalis*) have shown inhibitory activity against *P. viticola* under greenhouse and field conditions.^9^, ^16^ For example, a *M. officinalis* bark extract (honokiol and magnolol)^17^ and grapevine extracts (phenolic compounds)^18^, ^19^, ^20^, ^21^ decreased downy mildew severity under controlled and field conditions. Likewise, *Larix sp*. bark extracts showed efficacy against downy mildew under field conditions, with larixol and larixyl acetate as the main active compounds.^22^ Moreover, *S. officinalis* extracts decreased downy mildew severity and incidence on leaves and bunches under field conditions with an efficacy comparable to copper.^11^ *Salvia spp*. extracts contain several bioactive compounds belonging to classes of terpenoids and phenolic compounds.^23^, ^24^ For example, *Salvia spp*. terpenoids showed antifungal activity against *Candida albicans* (e.g., cryptotanshinone, dihydrotanshinone I, and tanshinone IIA),^25^ *Alternaria alternata*, *B. cinerea*, and *Fusarium oxysporum* (e.g., 11‐acetoxy carnosic acid, 11,12‐diacetoxy carnosic acid, and carnosic acid),^26^ antibacterial activity against *Bacillus cereus* (e.g., methyl carnosate),^27^ and antiviral activity (e.g., safficinolide and sageone).^28^ Moreover, rosmarinic acid is the most abundant phenolic compound of *Salvia spp*. with antimicrobial activity.^29^ A phytocomplex with high rosmarinic acid content, obtained from *S. officinalis* cultured cells, showed strong inhibitory activity against grapevine downy mildew, with greater efficacy compared to pure rosmarinic acid,^30^ suggesting that additional compounds contribute to anti‐oomycete activities of *Salvia spp*. extracts. Although the antimicrobial activity of *S. officinalis* extracts has been demonstrated, limited information is available on the bioactive compounds responsible for inhibiting *P. viticola*. Moreover, deeper knowledge is required on the antifungal properties of other sage species, due to the high economic value and production costs of *S. officinalis* for human consumption. For example, Russian sage (*Salvia yangii*; B. T. Drew; formerly *Perovskia atriplicifolia*) represents a promising candidate for the study of bioactive compounds due to its complex metabolic profile, which primarily includes terpenoids and phenolic compounds.^31^, ^32^, ^33^, ^34^, ^35^, ^36^ In addition to the relatively low economic value (e.g., lack of medicinal applications), Russian sage is appreciated for its rapid growth and tolerance to abiotic stresses, such as intense solar irradiation, large temperature fluctuations,^35^ semi‐arid habitats, prolonged drought, and soils with low fertility.^37^ Being successful over a wide range of climate and soil conditions, *S. yangii* has become popular and is cultivated worldwide for ornamental purposes,^32^ suggesting that this species could be a fast‐growing source of bioactive compounds. Despite the known antimicrobial activity of *Salvia spp*., the activity of Russian sage extracts against *P. viticola* was not investigated. This study aimed to characterize the efficacy of alcoholic extracts of Russian sage against grapevine downy mildew and to annotate bioactive compounds with inhibitory activity against *P. viticola*.

## 2 MATERIALS AND METHODS

### 2.1 Biological materials and growth conditions

Two‐year‐old plants of Russian sage (*S. yangii*; variety Blue Spire) were grown under field conditions in Northern Italy (San Michele all'Adige, Trento; GPS coordinates: 46.190848, 11.135700). Since the antimicrobial activities of *Salvia spp*. extracts can differ according to the tissue extracted,^38^, ^39^ shoots (whole aerial parts, including flowers, leaves, and stems), flowers, leaves, and stems were collected separately on 30th June 2023 to evaluate the potential differences in bioactivity of Russian sage extracts.

Two‐year‐old plants of the susceptible grapevine (*V. vinifera*) cultivar Pinot Noir grafted onto Kober 5BB were individually planted in 2.5 L pots containing a mixture of peat and pumice (3:1), and grown for 2 months in a greenhouse at 25 ± 1 °C with a photoperiod of 16 h light and relative humidity (RH) of 70 ± 10% as previously described.^40^

A *Plasmopara viticola* population was collected from an untreated vineyard in the Trentino region (Cultivar Pinot noir, northern Italy, N 46° 11′ 32.089″ and E 11° 8′ 15.997) and maintained on *V. vinifera* Pinot Noir plants by weekly inoculations under greenhouse conditions (25 ± 1 °C, photoperiod of 16 h light, and 70 ± 10% RH), as previously described.^40^ To obtain *P. viticola* inoculum, plants exhibiting disease symptoms were incubated overnight in a plastic humid chamber in the dark at 95 ± 5% RH and 25 ± 1 °C to promote pathogen sporulation, and sporangia were collected by washing the abaxial leaf surfaces bearing freshly sporulating lesions with cold (4 °C) distilled water, as previously described.^40^ The inoculum concentration was determined using a hemocytometer under a light microscope (H 600 LL FLUOR, Fluo Hund Wetz, Wetzlar, Germany), and the sporangial suspension was adjusted to 2.5 × 10^5^ sporangia/mL for the spray inoculation in leaf disk assays with alcoholic extracts of Russian sage, or to 5.0 × 10^5^ sporangia/mL to obtain a final inoculum concentration of 2.5 × 10^5^ sporangia/mL in the in the suspension used for drop inoculation in leaf disk assays with reference standards.

### 2.2 Alcoholic extract preparation from *Salvia yangii* plants

Shoot, flower, leaf, and stem samples of Russian sage were dried (35% of the fresh weight) under a chemical hood and crushed to obtain a fine powder using a mixer (HL1643, Philips, Netherlands). Alcoholic extracts of Russian sage samples were obtained from 20 g of dried powder of shoots [57% (w/w) leaves, 14% (w/w) flowers, and 29% (w/w) stems], flowers, leaves, or stems in 400 mL of 99.8% (v/v) ethanol using a Soxhlet extractor (Microglass Heim, Naples, Italy) as described for *S. officinalis* extracts.^11^ After 3 h of extraction, each extract was concentrated to dryness under reduced pressure (170 mbar) with a rotavapor (Laborota 4000/G3; Heidolph Instruments GmbH, Schwabach, Germany) at 57 °C. Each dried extract was resuspended in 20 mL (shoot and stem extracts) or 40 mL (flower and leaf extracts) of 99.8% (v/v) ethanol, and centrifuged at 5000 × *g* for 20 min at room temperature (Sorvall ST 16R, Thermo Scientific, Waltham, MA, USA) to obtain the alcoholic extract of each Russian sage sample with no sample loss during handling and resuspension, as previously reported for *S. officinalis* extracts^11^ (Supporting Information, Table S1). Different ethanol volumes were required to ensure complete solubilization of dried extracts from shoot and stem samples (20 mL) and from leaf and flower samples (40 mL), corresponding to dilutions of 1:1 and 1:2, respectively (Supporting Information, Table S1). Due to the different resuspension volumes, concentrations of Russian sage extracts were expressed as the weight (g) of dry biomass (shoot, flower, leaf, or stem samples) resuspended in 100 mL of treatment solution (10.0% ethanol and 0.5% DMSO in water; Supporting Information, Table S1). Alcoholic extracts of Russian sage were stored at −20 °C until further use.

### 2.3 Fractionation by preparative liquid chromatography

The shoot extract was fractionated using a preparative liquid chromatography (LC) instrument (Prep 150 LC System, Waters, Milford, MA, USA) equipped with a manual injector, a quaternary gradient pump (2545‐QGM), a UV/Visible detector (2489‐TUV), and a fraction collector (WFCIII). A C18 pre‐column (C18 column; particle diameter, 5 μm; pore dimension, 100 Å; column diameter 19 mm; and column length 100 mm; SunFire Prep, Waters) was followed by a C18 column (C18 OBD; particle diameter, 5 μm; pore dimension, 100 Å; column diameter 1.9 mm; and column length 100 mm; SunFire Prep, Waters) to achieve separation of analytes at room temperature (25 ± 2 °C). The injection solution was prepared by mixing 0.5 mL of shoot extract with 3.5 mL of 99.8% (v/v) methanol, 1.0 mL of dimethyl sulfoxide (DMSO, Sigma‐Aldrich, Merck, St. Louis, MO, USA), and 5.0 mL of water. The injection solution was vortexed (Vortex Genie 2, Scientific Industries, Bohemia, NY, USA), sonicated (Ultrasonic Cleaner USC1700TH, VWR, Milan, Italy) until resuspension, and filtered with a cellulose acetate filter (pore dimension, 0.2 μm; 16 534 K, Sartorius, Germany). An aliquot (5 mL) of the injection solution [consisting of 0.25 mL of shoot extract, 1.75 mL of 99.8% (v/v) methanol, 0.5 mL of DMSO, and 2.5 mL of water] was injected into the preparative LC instrument, and the mobile phases consisted of 0.1% formic acid in water (mobile phase A) and 0.1% formic acid in methanol (mobile phase B). The gradient program was set as follows: 10% of mobile phase B for 2 min, incremented to 90% of mobile phase B from 2 min to 18 min as elution gradient, and 90% of mobile phase B until 29 min as washing phase, followed by equilibration step for 5 min with 10% of mobile phase B, using a flow rate of 20 mL min^−1^. The shoot extract was fractionated by the preparative LC according to elution time (one fraction every 0.25 min), and 116 fractions of 5 mL each were collected. Six independent preparative LC runs were carried out, and each shoot extract fraction (5 mL) obtained from two LC runs was combined to limit variability. In particular, pools (pool of shoot extract fractions) of 10 fractions (10‐fraction pools; called for example F1‐10 and F11‐20 when combining LC fractions from the first to the tenth and from the eleventh to the twentieth of two LC runs, respectively), three fractions (3‐fraction pools; called for example F1‐3 and F4‐6 when combining LC fractions from the first to the third and from the fourth to the sixth of two LC runs, respectively), or 116 fractions (reconstructed shoot extract; called F1‐116 when combining all LC fractions from the first to the 116th of two LC runs) were obtained combining two independent preparative LC runs. Pool F1‐116 was concentrated to 40 mL under reduced pressure with a rotary evaporator (Rotavapor R‐210, Büchi, Flawil, Switzerland) at 57 °C, and each pool of shoot extract fractions was concentrated to 5 mL using a speed vacuum concentrator (Concentrator plus, Eppendorf, Hamburg, Germany) at 45 °C for 3 h and 60 °C for 3 h. Each pool of shoot extract fractions was then dried using a freeze dryer instrument (FreeZone 12, Labconco, Kansas City, MO, USA), resuspended in 0.5 mL of 99.8% (v/v) ethanol (corresponding to the shoot extract volume injected in two LC runs), and stored at −80 °C until further use.

### 2.4 Treatment of grapevine leaf disks with Russian sage extracts and inoculation with *Plasmopara viticola*

Each acholic extract of Russian sage (shoots, flowers, leaves, or stems) and each pool of shoot extract fractions was mixed with 99.8% (v/v) ethanol, DMSO, and water to obtain the treatment solution (acholic extract in 10.0% ethanol and 0.5% DMSO; Supporting Information, Table S1). Different concentrations of shoot extracts [0.5%, 1.0%, 2.5%, or 5.0% (w/v)] were tested. Leaf, flower, and stem extracts were used at 1.0% (w/v), and each pool of shoot extract fractions was used at 5.0% (w/v). Concentrations of alcoholic extracts and shoot extract fractions were expressed as the weight (g) of dry biomass (shoot, flower, leaf, or stem samples) resuspended in 100 mL of treatment solution (10.0% ethanol and 0.5% DMSO in water) after extraction, fractionation, drying, and resuspension, and the dosage of 5.0% (w/v) corresponded to 5.0% (v/v) of resuspended alcoholic extract as previously reported for *S. officinalis* extracts,^11^ according to the volumes reported in Supporting Information, Table S1. Copper hydroxide (2 g L^−1^ COPRANTOL HI BIO, Syngenta, Switzerland) and a solution of 10.0% ethanol and 0.5% DMSO in water (Control) were used in each experiment as reference fungicide and control, respectively.

Leaf disks (19 mm diameter) were obtained from the greenhouse‐grown grapevine plants (from the third and fourth leaves from the apical meristem) with a cork borer, and they were placed randomly on four layers of wet filter paper in dishes (90 mm diameter; five leaf disks for each dish) with the abaxial surface uppermost.^41^ Treatments were applied to the abaxial surface of each leaf disk using a hand sprayer (1 mL for each plate), and leaf disks were dried under a laminar flow hood for 10 min at room temperature.^42^ Leaf disks were inoculated with the *P. viticola* suspension (2.5 × 10^5^ sporangia/mL) using a hand sprayer (1 mL for each plate; spray inoculation), incubated in the dark at 25 ± 1 °C overnight, dried under a chemical hood, and incubated for 6 days under greenhouse conditions (25 ± 1 °C, photoperiod of 16 h light, and 70 ± 10% RH) to allow downy mildew development.^41^ Downy mildew severity was assessed visually on each leaf disk at 7 days post inoculation (dpi) as a percentage of the leaf disk surface covered by sporulation according to the guidelines of the European and Mediterranean Plant Protection Organization (EPPO).^43^ The disease severity of each replicate (dish) was calculated as the average of the disease severity of leaf disks contained in the dish. The disease reduction (efficacy) was calculated for each replicate according to the following equation: (disease severity of control leaf disks ‐ disease severity of treated leaf disks) / disease severity of control leaf disks × 100. Phytotoxic effects were assessed visually by checking for discoloration, chlorosis, and whitening of leaf disks, and expressed as the percentage of the leaf disk surface affected by phytotoxicity according to the EPPO guidelines.^44^ For each trial (i.e., alcoholic extracts of Russian sage or pools of shoot extract fractions), five replicates (dishes with five leaf disks each) were assessed for each treatment, and the experiment was carried out twice in two different weeks within 2 months, for a total of 50 leaf disks per treatment. Active and non‐active pools of shoot extract fractions were selected according to the efficacy against *P. viticola* (selected fractions), and they were analyzed by gas chromatography–mass spectrometry (GC–MS) and ultra‐high‐pressure liquid chromatography‐high resolution mass spectrometry (UHPLC‐HRMS).

### 2.5 Metabolomic analysis by gas chromatography–mass spectrometry (GC–MS)

An aliquot (2.5 μL) of each selected fraction was dried using a speed vacuum concentrator (Concentrator plus, Eppendorf) at 45 °C for 20 min and resuspended in 62.5 μL of 99.5% (v/v) acetone. Samples were analyzed with a GC–MS instrument (QP2010 SE, Shimadzu, Kyoto, Japan) equipped with an autosampler, a split injection port, a gas chromatography oven, and a single quadrupole mass spectrometer. Chromatographic separation of analytes was obtained with a low‐polarity SLB‐5 MS column (film thickness, 0.25 μm; column diameter, 0.25 mm; and column length, 30 m; Supelco, Bellefonte, PA, USA). For GC–MS analysis, 1 μL of each sample was injected using a split ratio of 1:10, and helium was used as carrier gas with a flow rate of 1 mL min^−1^. The temperature of the chromatographic run was kept at 40 °C for 5 min, then increased to 280 °C with a rate of 10 °C min^−1^ and held at 280 °C for 10 min. The injector temperature and transfer line temperature were maintained at 280 °C, while the ion source temperature was set at 230 °C with 70 eV as ionization potential. Mass spectrometry (MS) data were acquired in full scan mode with a mass‐to‐charge ratio (*m/z*) range between 40 and 500.

Full scan MS data of the selected fractions of Russian sage were processed using GC–MS solution software (Shimadzu, Kyoto, Japan) to annotate features detected in all active fractions, but not detected in non‐active fractions, according to visual observation of chromatograms. Compound annotation was carried out by comparing full scan MS data to reference spectra of NIST 2017 database (https://chemdata.nist.gov/dokuwiki/doku.php?id=chemdata:nist17) to assign the putative chemical formula and putative chemical name, imposing a minimum spectral similarity of 75% (annotated compounds). Annotated compounds were classified into putative chemical classes according to the classification obtained with the NP classifier web‐based application (https://npclassifier.ucsd.edu/) by SMILES structure search.^45^

### 2.6 Metabolomic analysis by ultra‐high‐pressure liquid chromatography‐high resolution mass spectrometry (UHPLC‐HRMS)

An aliquot (1 μL) of each selected fraction was diluted in 249 μL of 99.8% (v/v) methanol and 250 μL of water to obtain a methanol:water (50:50; v/v) solution. Samples were centrifuged at 21 000 × *g* for 5 min at 4 °C (Centrifuge 5810 R, Eppendorf), and UHPLC‐HRMS analysis was carried out using a Vanquish Flex UHPLC System coupled with an Orbitrap Exploris 240 mass spectrometer (Thermo Scientific). A Waters Acquity HSS T3 C18 column (particle diameter, 1.8 μm, column diameter 2.1 mm, column length 150 mm; Waters) was used to separate analytes in a thermostatted column compartment at 35 °C. The mobile phases consisted of 0.1% formic acid in water (mobile phase A) and 0.1% formic acid in acetonitrile (mobile phase B). The chromatographic separation was performed as reported by Avesani *et al*. (2025).^46^ Briefly, 20 μL of each sample was injected, and a linear gradient program was set as follows: 5% of mobile phase B in the first 1 min, incremented to 99% of mobile phase B from 1 min to 14 min, held to 99% of mobile phase B until 19 min, followed by decrement from 99% to 5% of mobile phase B until 19.5 min, and 5% of mobile phase B until 26 min, with a flow rate of 0.4 mL min^−1^. The ion source parameters were set as follows: spray voltage was 3500 V in positive heated electrospray ionization (HESI) mode and 2500 V in negative HESI mode; the temperature of ion source, capillary, and auxiliary gas was 350 °C, 300 °C, and 300 °C, respectively; the settings of sheath gas, aux gas, and sweep gas were 50 arb, 10 arb, and 0 arb, respectively; the S‐lens RF value was 70 V; automatic gain control target was set as custom (100%); and the maximum injection time was 100 ms. Mass spectrometric conditions were set according to Avesani *et al*. (2025),^46^ and the *m/z* ranged from 90 to 1350 with a resolution of 90 000 for the full scan mass spectrometry (MS) analysis and 45 000 for data‐dependent tandem mass spectrometry (ddMS^2^) fragmentation analysis. ddMS^2^ fragmentation was acquired separately in each ionization mode at 20, 40, and 60 normalized collision energies with a 2 *m/z* isolation window.

Full scan MS data and ddMS^2^ fragmentation data were processed using an untargeted metabolomics workflow on the Compound Discoverer software (Version 3.3 SP2; Thermo Scientific) that included spectrum selection from raw data, chromatographic alignment between multiple spectra, extraction of mass chromatograms, feature detection according to retention time and *m/z*, peak area normalization, and prediction of chemical composition (Supporting Information, Fig. S1). The molecular weight, predicted chemical formula, delta mass error between the measured molecular weight and the molecular weight calculated from the predicted chemical formula (with a maximum accepted mass error of 5 ppm), reference ion, and fragmentation information were obtained for each feature. Moreover, the Compound Discoverer workflow allowed feature annotation (Supporting Information, Fig. S1) by searching the ddMS^2^ of each feature in mzCloud (https://www.mzcloud.org/), and by searching molecular weight and predicted chemical formula in ChemSpider database (https://www.chemspider.com/) and in Mass Lists database, which included Human Metabolome Database (https://hmdb.ca/), Lipid Maps (https://www.lipidmaps.org/), MassBank (https://massbank.eu/MassBank/), FooDB (https://foodb.ca/), PlantCyc (https://www.plantcyc.org/), ChEBI (https://www.ebi.ac.uk/chebi/), Phenol‐Explorer (http://phenol-explorer.eu/), and Arita lab (http://metabolomics.jp/wiki/). The ChemSpider database and Mass Lists database were used to maximize feature annotation as previously described.^46^

Features of interest were selected using Compound Discoverer software by imposing an abundance ratio greater than two in pairwise comparisons between active and non‐active fractions against *P. viticola*, while ensuring low variability among active fractions with an abundance ratio lower than two in the pairwise comparisons among active fractions. Molecular weight and predicted chemical formula of the 100 most abundant features (50 and 50 features with the highest peak area detected in positive HESI mode and negative HESI mode, respectively) were searched in the PubChem (https://pubchem.ncbi.nlm.nih.gov/), LOTUS (https://lotus.naturalproducts.net/), PlantaeDB (https://plantaedb.com/), and KNApSAcK (https://www.knapsackfamily.com/KNApSAcK/) databases to retrieve the putative chemical name and chemical class. Database reference spectra and *in silico* fragmentation spectra obtained with Compound Discoverer software were visually compared with the experimental full scan MS spectra and ddMS2 fragmentation spectra to confirm the putative chemical name of the most abundant features (annotated compounds). Annotated compounds were classified into 13 putative chemical classes (cyclic polyketides, diterpenoids, fatty acyl glycosides, fatty esters, glycerophospholipids, meroterpenoids, monoterpenoids, naphthalenes, ornithine alkaloids, phenylpropanoids, sesquiterpenoids, triterpenoids, and unknown), according to classification obtained with the NP classifier web‐based application (https://npclassifier.ucsd.edu/) by SMILES structure search.^45^

### 2.7 Validation of reference standard by ultra‐high‐pressure liquid chromatography‐high resolution mass spectrometry analysis and activity assays against *Plasmopara viticola*

Five terpenoids were selected according to their abundance in active fractions, compound availability, and compound cost, and reference standards were used to validate the annotation of 7‐methylrosmanol (Sigma‐Aldrich, Merck, St. Louis, Missouri, USA), 12‐O‐methylcarnosic acid (PhytoLab, Vestenbergsgreuth, Germany), carnosic acid (Cayman, Ann Arbor, Michigan, USA), carnosol (Apollo Scientific, Manchester, UK), and maslinic acid (TRC, Burlington, Ontario). Each reference standard was dissolved in 99.8% (v/v) methanol and analyzed by UHPLC‐HRMS at a concentration of 10 g L^−1^ in methanol:water (50:50; v/v) using the protocol described above for selected fractions of Russian sage. For compound identification, retention time, full scan MS spectra, and ddMS^2^ fragmentation spectra of annotated compounds were compared with those of the respective reference standard. Chemical structures were generated using the ACD/ChemSketch software (ACD/Labs, Toronto, Ontario, Canada), according to reference standard information.

Different concentrations of reference standards were tested against *P. viticola* (1.0, 5.0, 10.0, and 100.0 mg L^−1^) in leaf disk assays. Drop inoculation was used to reduce the amount of reference standard required, rather than spray inoculation. Each reference standard was dissolved in 99.8% (v/v) ethanol and DMSO to obtain a two‐fold concentrated treatment solution (350 μL). Each two‐fold concentrated treatment solution was mixed with 350 μL of the *P. viticola* suspension (5.0 × 10^5^ sporangia/mL; two‐fold concentrated inoculum suspension), and the resulting mixture was used for drop inoculation of leaf disks (i.e., inoculation suspension containing the reference standard at the appropriate concentration and 2.5 × 10^5^ sporangia/mL in 10% ethanol and 0.5% DMSO). As a control, the *P. viticola* suspension (2.5 × 10^5^ sporangia/mL) was prepared in 10% ethanol and 0.5% DMSO in water (Control). Leaf disks (19 mm diameter) were obtained from the greenhouse‐grown grapevine plants as described above, and five drops (5 μL) of each suspension were applied to the abaxial surface of each leaf disk (for drop inoculation). Leaf disks were incubated in the dark at 25 ± 1 °C overnight, dried under a chemical hood, and incubated for 6 days under greenhouse conditions (25 ± 1 °C, photoperiod of 16 h light, and 70 ± 10% RH). Downy mildew severity was assessed visually on each leaf disk at 7 dpi as a percentage of the leaf disk surface covered by sporulation according to the EPPO guidelines,^43^ calculated as the sum of the five inoculum drops: 0%, no sporulation; 10%, scarce sporulation; 20%, dense sporulation.^47^ The disease severity of each replicate (dish), disease reduction, and phytotoxic effects were calculated as described above. Five replicates (dishes with five leaf disks each) were assessed for each treatment, and the experiment was carried out twice in two different weeks within 2 months, for a total of 50 leaf disks per treatment.

### 2.8 Statistical analysis

Disease severity and efficacy data were analyzed using Past 4.03 software (https://www.nhm.uio.no/english/research/resources/past/). Normality (Shapiro test, *P* > 0.05) and homogeneity of variance (Levene test, *P* > 0.05) were assessed. Since the assumptions of parametric tests were not met, the non‐parametric Kruskal–Wallis test was used to assess consistency among experimental repetitions (*P* > 0.05). For each disease assessment, data from the two experiments were pooled, and a Kruskal–Wallis test followed by Mann–Whitney *post hoc* test was used to identify significant differences among treatments (*P* ≤ 0.05).

## 3 RESULTS

### 3.1 Alcoholic extracts of Russian sage decreased downy mildew severity on grapevine leaf disks

**Figure 1:**
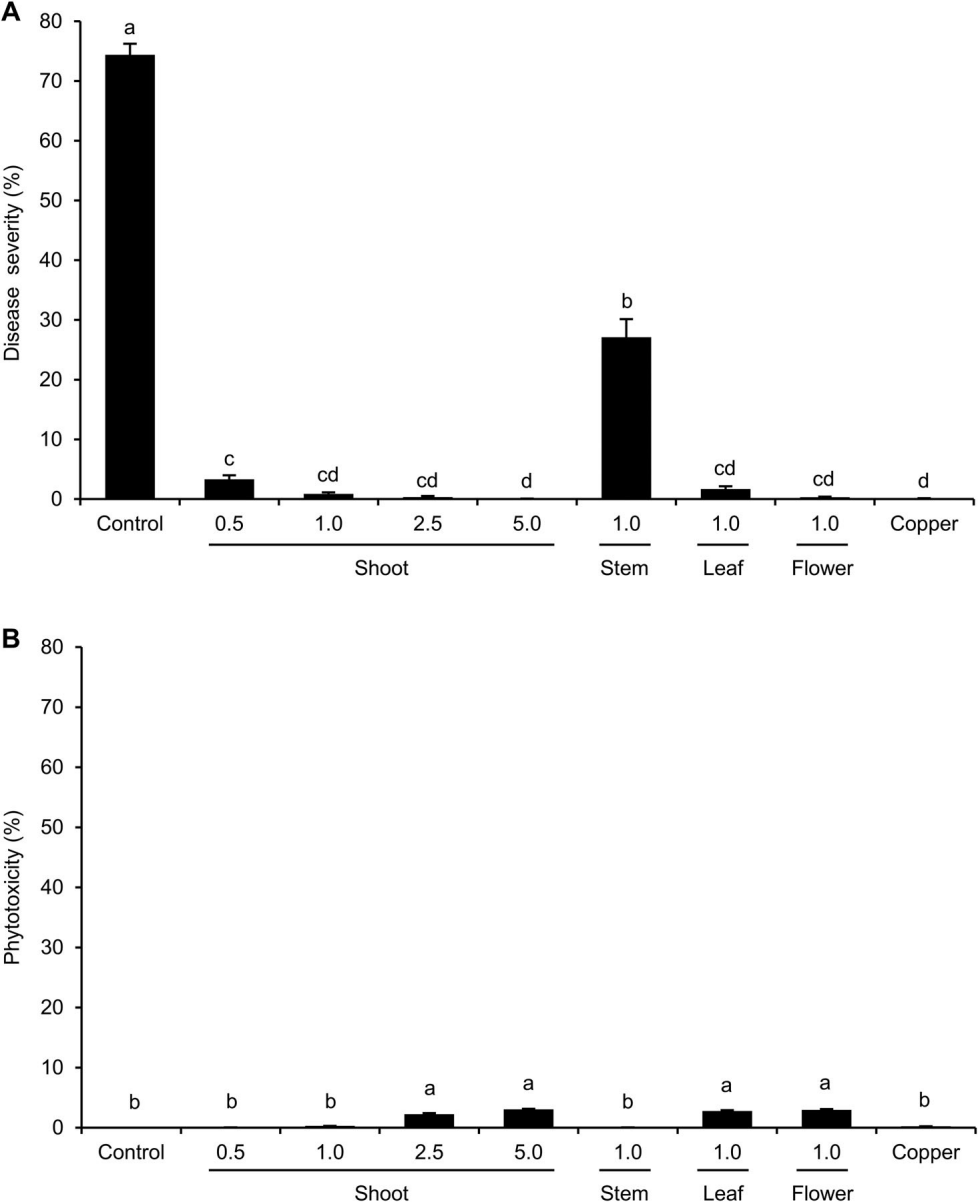
Alcoholic extracts of Russian sage decreased downy mildew severity. Leaf disks were treated with water (Control), 0.5%, 1.0%, 2.5%, or 5.0% (w/v) shoot extract, 1.0% (w/v) stem extract, 1.0% (w/v) leaf extract, and 1.0% (w/v) flower extract of Russian sage in a treatment solution containing 10% ethanol and 0.5% DMSO. Copper hydroxide (Copper) was applied as a reference fungicide. Downy mildew severity (A) and phytotoxic effects (B) were assessed visually as the percentage of leaf disk area with disease symptoms (downy mildew sporulation) and phytotoxicity (discoloration, chlorosis, and whitening) at 7 days post inoculation (dpi), respectively. Comparable results were obtained between the two experiments (Kruskal–Wallis *P* > 0.05), and data were pooled. Mean and standard error values of ten replicates (dishes with five leaf disks each) from the two experiments are reported for each treatment. Different letters indicate significant differences among treatments, according to the Kruskal–Wallis test with Mann–Whitney *post hoc* test (*P* ≤ 0.05).

Alcoholic extracts of Russian sage were obtained from shoot, flower, leaf, and stem samples using a Soxhlet extractor, and they decreased downy mildew severity on grapevine leaf disks (Fig. 1(A)). The efficacy of treatments with 0.5%, 1.0%, 2.5%, and 5.0% (w/v) shoot extract [expressed as the weight (g) of dry biomass (shoot, flower, leaf, or stem samples) resuspended in 100 mL of treatment solution (10.0% ethanol and 0.5% DMSO in water) after extraction, drying, and resuspension, with the dosage of 5.0% (w/v) corresponding to 5.0% (v/v) of resuspended alcoholic extract; Supporting Information, Table S1) was 96 ± 1%, 99 ± 1%, 99 ± 1%, and 99 ± 1% (mean ± standard error), respectively. The disease severity of leaf disks treated with 1.0%, 2.5%, and 5.0% shoot extract was comparable to that obtained with copper, which reached an efficacy of 99 ± 1%. Treatments with 1.0% leaf and 1.0% flower extracts decreased downy mildew severity with an efficacy of 98 ± 1% and 99 ± 1%, respectively, while those with 1.0% stem extract were partially active (64 ± 4% efficacy). Negligible phytotoxic effects were observed on leaf disks treated with 1.0% flower, 1.0% leaf, 2.5% shoot, and 5% shoot extracts, and no phytotoxicity was detected on leaf disks treated with copper, 0.5% shoot, 1.0% shoot, and 1.0% stem extracts (Fig. 1(B)).

**Figure 2:**
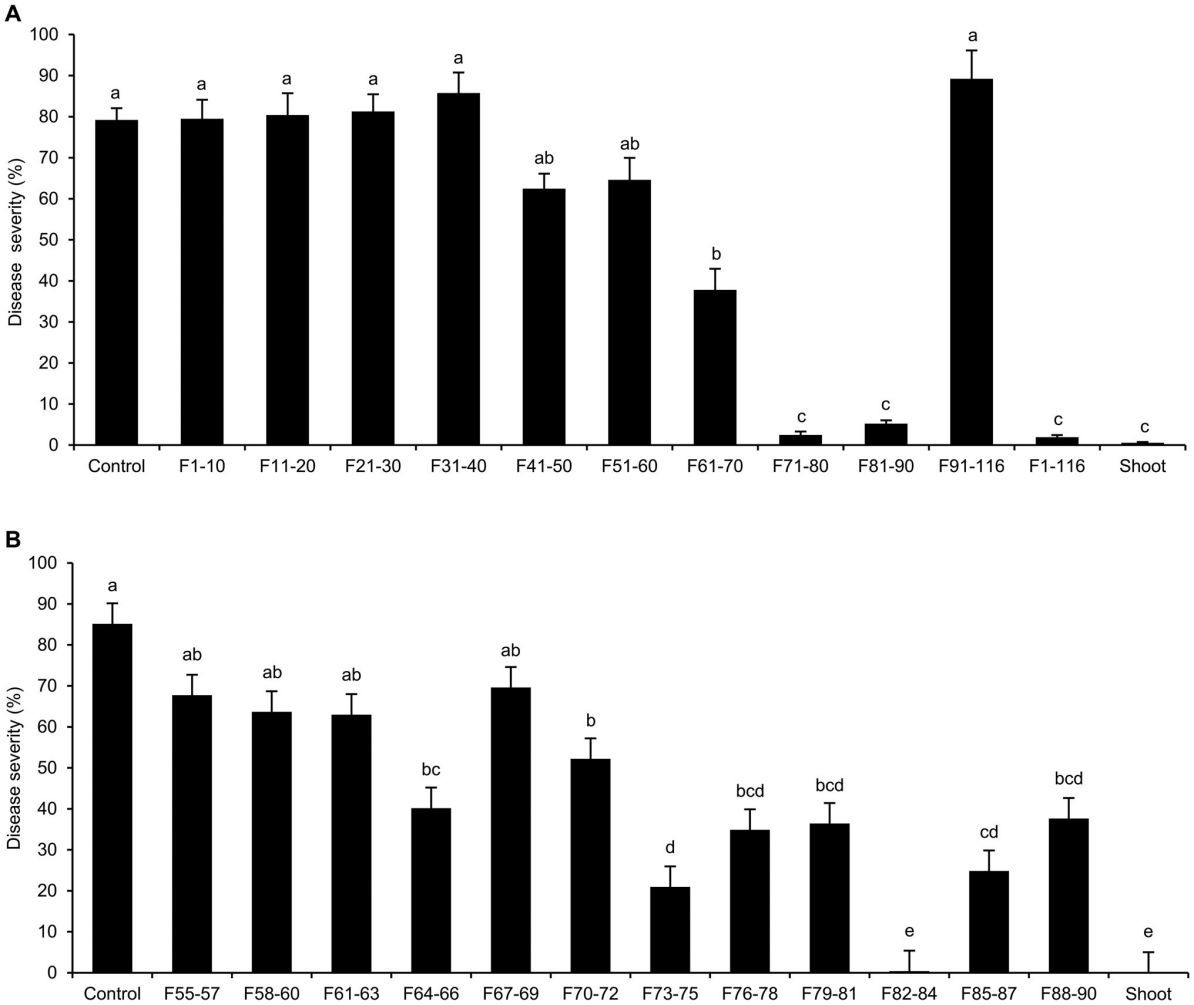
Shoot extract fractions of Russian sage decreased downy mildew severity. Leaf disks were treated with water (Control) or 5% (w/v) of each pool of shoot extract fractions obtained by preparative liquid chromatography (LC) and combined into pools of ten fractions (A; from F1‐10 to F91‐116; called F1‐10 when combining LC fractions from the first to the tenth), three fractions (B; from F55‐57 to F88‐90; called F55‐57 when combining LC fractions 55th, 56th, and 57th), or 116 fractions (reconstructed shoot extract obtained combining all LC fractions; F1‐116) in a treatment solution containing 10% ethanol and 0.5% DMSO. As a control, leaf disks were treated with 5% (w/v) shoot extract. Downy mildew severity was assessed visually as the percentage of leaf disk area with disease symptoms (downy mildew sporulation) at 7 days post inoculation (dpi). No phytotoxic effects were observed for all treatments, except for the treatment with 5% shoot extract (3 ± 1% phytotoxicity). Comparable results were obtained between the two experiments (Kruskal–Wallis *P* > 0.05), and data were pooled. Mean and standard error values of ten replicates (dishes with five leaf disks each) from the two experiments are reported for each treatment. Different letters indicate significant differences among treatments, according to the Kruskal–Wallis test with Mann–Whitney *post hoc* test (*P* ≤ 0.05).

To annotate bioactive compounds present in all aerial tissues of Russian sage, the shoot extract (flowers, leaves, and stems) was fractionated using preparative LC, and aliquots of the resulting fractions were combined into pools of either 10 fractions (10‐fraction pools; called F1‐10 when combining LC fractions from the first to the tenth) or three fractions (3‐fraction pools; called F55‐57 when combining LC fractions 55th, 56th, and 57th) for activity assays against *P. viticola* at a concentration of 5% (w/v; expressed as the weight of dry biomass of shoot samples resuspended in the treatment solution after extraction, drying, resuspension, and fractionation; Supporting Information, Table S1), which corresponds to the maximum‐efficacy dosage of shoot extracts. Treatments with the 10‐fraction pools F61‐70, F71‐80, and F81‐90 decreased downy mildew severity with efficacies of 52 ± 7%, 97 ± 1%, and 93 ± 1%, respectively (Fig. 2(A)). In particular, downy mildew severity was comparable on leaf disks treated with fraction pools F71‐80, F81‐90, reconstructed shoot extract (F1‐116), and shoot extract (Fig. 2(A)). Conversely, treatments with fraction pools F1‐10, F11‐20, F21‐30, F31‐40, F41‐50, F51‐60, and F91‐116 did not decrease downy mildew severity compared to the control (Fig. 2(A)). To better characterize the active fractions of the shoot extract, 3‐fraction pools were tested in leaf disk assays, and fraction pools F64‐66, F70‐72, F73‐F75, F76‐78, F79‐81, F82‐84, F85‐87, and F88‐90 decreased downy mildew severity with no phytotoxic effects (Fig. 2(B)). In particular, downy mildew severity was comparable on leaf disks treated with fraction pools F82‐84 (99 ± 1% efficacy) and 5% shoot extract, possibly due to the presence of bioactive compounds (Fig. 2(B)).

### 3.2 Active fractions of shoot extracts contained putative bioactive terpenoids against grapevine downy mildew

Active fractions (F1‐116, F81‐90, and F82‐84) against *P. viticola* were analyzed by GC–MS and UHPLC‐HRMS to annotate potential bioactive compounds. Non‐active fractions (F1‐10 and F91‐116) were also analyzed to rule out any potential background signals arising from solvent impurities or reagents used during extraction and fractionation, and to exclude inactive compounds with different polarity (F1‐10) or comparable polarity (F91‐116) to those in the active fractions. In the GC–MS analysis, 13 features were detected in all active fractions (F1‐116, F81‐90, and F82‐84) and not detected in non‐active fractions (F1‐10 and F91‐116), and six of them were annotated as putative diterpenoids [(+/−)‐demethylsalvicanol and isocarnosol (two features)], fatty acids (nonanoic acid), oxaspiro compounds [7,9‐di‐tert‐butyl‐1‐oxaspiro(4,5)deca‐6,9‐diene‐2,8‐dione], and steroids (γ‐sitosterol) (Supporting Information, Table S2). Annotated compounds by GC–MS analysis were previously found in *Salvia spp*. extracts according to database search, except for 7,9‐di‐tert‐butyl‐1‐oxaspiro(4,5)deca‐6,9‐diene‐2,8‐dione (Supporting Information, Table S2).

**Figure 3:**
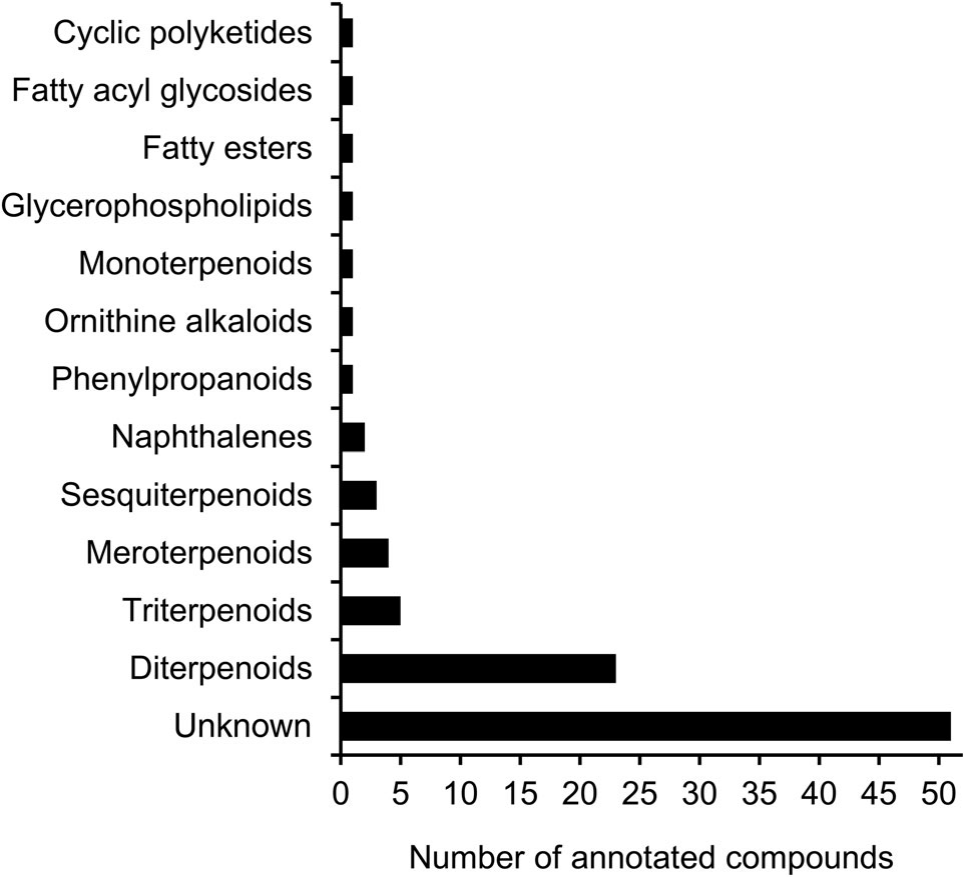
Classification of annotated compounds detected in shoot extract fractions of Russian sage. The numbers of annotated compounds detected by ultra‐high‐pressure liquid chromatography‐high resolution mass spectrometry (UHPLC‐HRMS) analysis in active fractions of shoot extract are reported for each chemical class. Five annotated compounds [(+)‐(1*R*)‐1,12‐dihydroxy‐20‐norabieta‐5 (10),8,11,13‐tetraene; carnosol; maslinic acid; pisiferdiol; and virgatic acid] were detected in positive and negative HESI mode.

In the UHPLC‐HRMS analysis, features of interest were selected by imposing an abundance ratio greater than two in the pairwise comparisons between active and non‐active fractions, while ensuring low variability among active fractions (abundance ratio lower than two). Annotation of the 100 most abundant features detected in active fractions allowed the classification of 44 annotated compounds into 13 putative chemical classes, and five of them were detected in both positive and negative HESI mode [(+)‐(1*R*)‐1,12‐dihydroxy‐20‐norabieta‐5 (10),8,11,13‐tetraene; carnosol; maslinic acid; pisiferdiol; and virgatic acid] (Fig. 3 and Supporting Information, Table S3). However, 51 most abundant features were not annotated and were classified as unknown. The majority of annotated compounds belonged to the class of putative terpenoids, such as 23 diterpenoids, five triterpenoids, four meroterpenoids, three sesquiterpenoids, and one monoterpenoid (Supporting Information, Table S3). Moreover, the other compounds were annotated as putative cyclic polyketides, fatty acyl glycosides, fatty esters, glycerophospholipids, naphthalenes, ornithine alkaloids, and phenylpropanoids (Supporting Information, Table S3).

**Figure 4:**
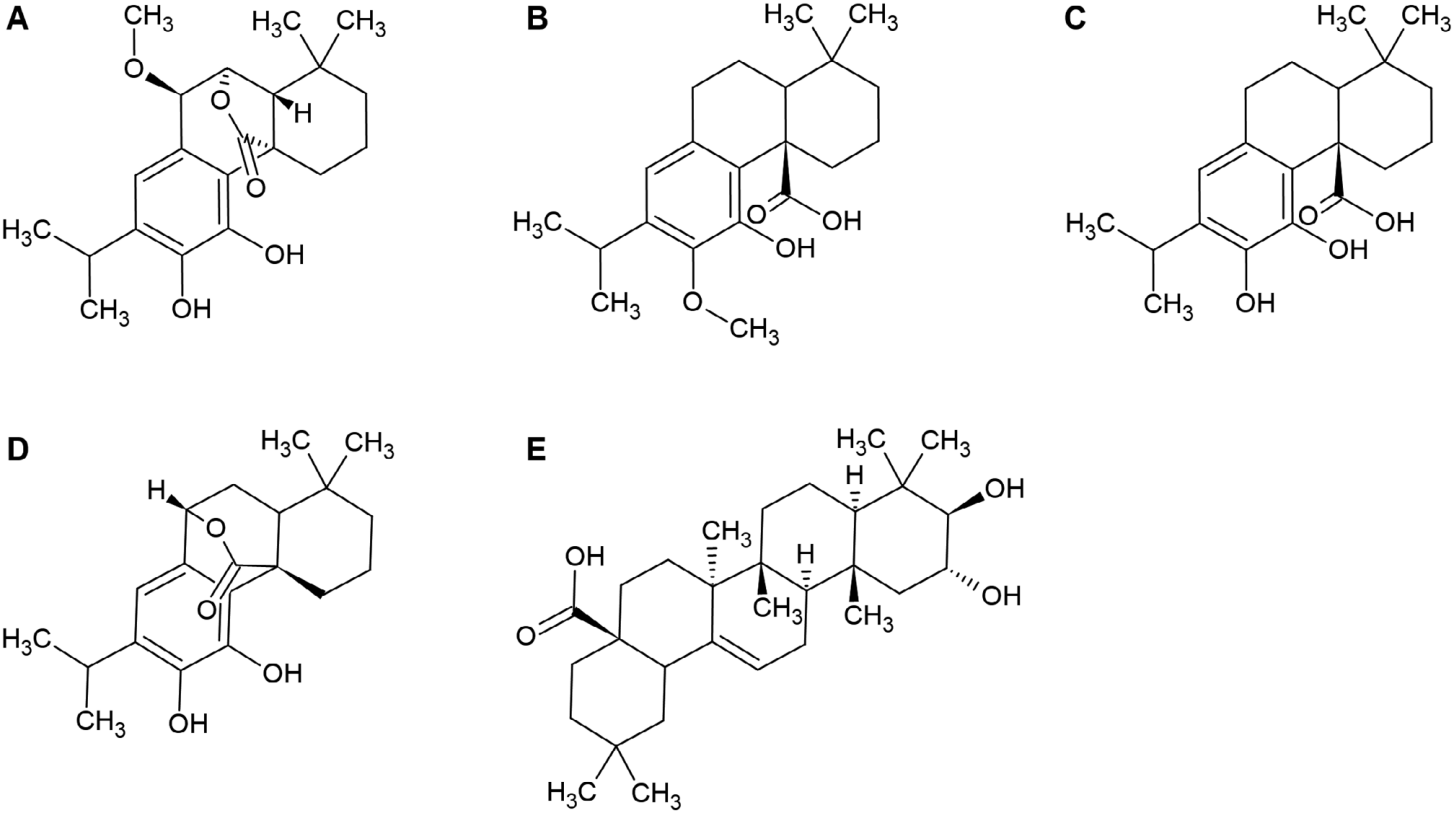
Chemical structure of bioactive terpenoids detected in shoot extract fractions of Russian sage. Chemical structures of 7‐methylrosmanol (A), 12‐O‐methylcarnosic acid (B), carnosic acid (C), carnosol (D), and maslinic acid (E) detected in alcoholic extracts of Russian sage were generated according to reference standard information.

**Figure 5:**
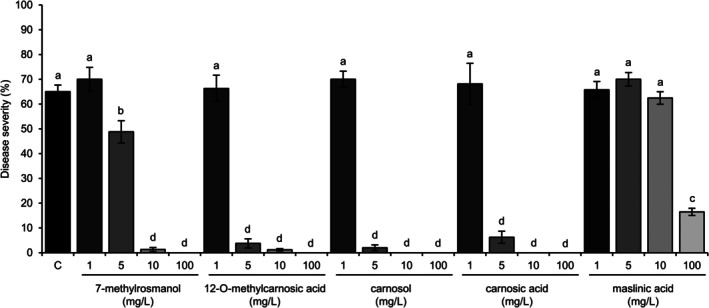
Pure compounds of Russian sage decreased downy mildew severity. Leaf disks were inoculated with a *P. viticola* suspension in water (Control, C) or different concentrations (1.0, 5.0, 10.0, and 100.0 mg L^−1^) of 7‐methylrosmanol, 12‐O‐methylcarnosic acid, carnosic acid, carnosol, and maslinic acid in 10% ethanol and 0.5% DMSO. Downy mildew severity was assessed on each leaf disk at 7 days post inoculation (dpi) as a percentage of the leaf disk surface covered by sporulation, calculated as the sum of the five inoculum drops: 0%, no sporulation; 10%, scarce sporulation; 20%, dense sporulation. No phytotoxic effects were observed for all treatments. Comparable results were obtained between the two experiments (Kruskal–Wallis *P* > 0.05), and data were pooled. Mean and standard error values of 10 replicates (dishes with five leaf disks each) from the two experiments are reported for each treatment. Different letters indicate significant differences among treatments, according to the Kruskal–Wallis test with Mann–Whitney *post hoc* test (*P* ≤ 0.05).

Five terpenoids were selected according to their abundance in active fractions (Fig. 4 and Supporting Information, Table S3), and their identification was confirmed by UHPLC‐HRMS analysis using reference standards, namely 7‐methylrosmanol, 12‐O‐methylcarnosic acid, carnosic acid, carnosol, and maslinic acid (Supporting Information, Fig. S2), and these terpenoids decreased downy mildew symptoms on grapevine leaf disks when applied as pure compounds (Fig. 5). In particular, treatments with 7‐methyrosmanol, 12‐O‐methylcarnosic acid, carnosol, and carnosic acid decreased downy mildew severity with high efficacy (> 98%) and no phytotoxic effects when applied at concentrations of 10.0 mg L^−1^ or 100.0 mg L^−1^ in the *P. viticola* suspension. Likewise, strong disease reduction was observed in leaf disks inoculated with a *P. viticola* sporangial suspension containing 5.0 mg L^−1^ 12‐O‐methylcarnosic acid (93 ± 3% efficacy), 5.0 mg L^−1^ carnosol (97 ± 2% efficacy), 5.0 mg L^−1^ carnosic acid (90 ± 4% efficacy), and 100.0 mg L^−1^ maslinic acid (74 ± 2% efficacy) with no phytotoxic effects. Moreover, 5.0 mg L^−1^ 7‐methyrosmanol (25 ± 7% efficacy) partially decreased downy mildew symptoms, whereas no disease reduction was observed in leaf disks inoculated with a *P. viticola* sporangial suspension containing 1.0 mg L^−1^ of the tested compounds.

## 4 DISCUSSION

Given its complex metabolic composition^31^, ^32^, ^33^, ^34^, ^35^ and adaptability to a wide range of climate and soil conditions,^32^, ^35^ Russian sage represents a promising fast‐growing source of bioactive compounds. In our study, alcoholic extracts of Russian sage demonstrated strong efficacy against downy mildew in grapevine leaf disk assays, with no phytotoxic effects, highlighting their potential as plant‐derived products for the further development of sustainable alternatives for grapevine protection. Previous studies showed inhibitory activities of 5% (v/v) *S. officinalis* alcoholic extracts^11^ and 0.5% (w/v) *S. officinalis* phytocomplex^30^ against grapevine downy mildew under controlled and field conditions. Similarly, 10% (v/v) *S. officinalis* and 10% (v/v) *S. rosmarinus* (formerly *Rosmarinus officinalis*) leaf extracts inhibited the germination of *Phytophthora spp*. zoospores (e.g., *P. capsica*, *P. megakarya*, and *P. palmivora*),^48^ highlighting the broad anti‐oomycete potential of *Salvia spp*. extracts. Here, we found that 1% (w/v) flower extract, 1% (w/v) leaf extract, and 1% (w/v) shoot extract, which was composed of 57% (w/w) leaves, 14% (w/w) flowers, and 29% (w/w) stems, decreased downy mildew severity with an efficacy comparable to that of copper (99 ± 1% efficacy). Conversely, 1% (w/v) stem extract was only partially active (64 ± 4% efficacy), indicating that bioactive compounds are primarily available in *S. yangii* leaves and flowers and that stems can be excluded from plant harvest to allow rapid recovery and shoot regrowth after cutting. Likewise, the antibacterial and antifungal activities of *S. hispanica* methanolic extracts differed according to the plant tissue (e.g., seeds, sprouts, leaves, flowers, roots, and herbs), with leaf extracts exhibiting the highest inhibitory activity within the concentration range of 0.125–1.0% (w/v).^38^ Moreover, leaf extracts of *S. officinalis* have been reported to exhibit stronger antimicrobial activity compared to flower or stem extracts within the concentration range of 0.6–12.0% (v/v),^39^ corroborating tissue‐specific accumulation of bioactive compounds.

To further investigate the active components of the alcoholic extracts of Russian sage, shoot extracts were fractionated using preparative LC, the activity of the resulting fractions was assessed against *P. viticola*, and untargeted GC–MS and UHPLC‐HRMS analyses were used to annotate bioactive compounds. Hydrophobic fractions of preparative LC (e.g., F71‐80, F81‐90, and F82‐84) showed high efficacy against downy mildew, suggesting that compound polarity may influence the anti‐oomycete activity. Although F71‐80 and F81‐90 fraction pools showed comparable disease reduction, the efficacy of F82‐84 was higher than that of F70‐72, F73‐75, F76‐78, and F79‐81, suggesting synergistic effects among the bioactive compounds in the F71‐80 fraction pool. In the GC–MS analysis of the active fractions, two diterpenoids, one fatty acid, one oxaspiro compound, one sterol, and seven unknown compounds were found. Moreover, the most abundant features detected in active fractions by UHPLC‐HRMS analysis were annotated as 23 diterpenoids, five triterpenoids, four meroterpenoids, three sesquiterpenoids, one monoterpenoid, one cyclic polyketide, one fatty acyl glycoside, one fatty ester, one glycerophospholipid, two naphthalenes, one ornithine alkaloid, and one phenylpropanoid, indicating the complementarity of GC–MS and UHPLC‐HRMS analysis to improve compound annotation. Likewise, *S. yangii* is known to contain a complex array of diterpenoids, triterpenoids, monoterpenoids, and sesquiterpenoids.^31^, ^32^, ^33^, ^34^, ^35^ In particular, compounds detected in active fractions of shoot extracts have been previously identified in Russian sage, such as carnosic acid,^31^, ^32^, ^35^ carnosol,^31^, ^32^ miltirone,^32^ 7‐methylrosmanol, and its isomer 11‐methylrosmanol,^31^, ^32^ indicating consistency of the metabolic profiles. Moreover, some compounds found in active fractions of shoot extracts are known for their antimicrobial properties, such as bakuchiol against *Clavibacter michiganensis* and *Pseudomonas syringae*,^49^ butylphthalide against *C. albicans*,^50^ mansonone C against *Phytophthora parasitica*,^51^ menadione against *Paenibacillus larvae*,^52^ salvipisone against *B. subtilis*, *Enterococcus faecalis*, *Escherichia coli*, *Klebsiella pneumoniae*, and *Staphylococcus aureus*,^53^ and zaluzanin D against *B. cinerea*, *Curvularia lunata*, *Colletotrichum lindemuthianum*, *F. equisetii*, *F. oxysporum* and *R. solani*,^54^ suggesting their possible contribution against *P. viticola*. In particular, the analysis of reference standards confirmed the identification of four diterpenoids (7‐methylrosmanol, 12‐O‐methylcarnosic acid, carnosic acid, and carnosol), and one triterpenoid (maslinic acid), which decreased downy mildew severity in leaf disk assays in a dose‐dependent manner. Among them, 7‐methyrosmanol, 12‐O‐methylcarnosic acid, carnosol, and carnosic acid demonstrated strong efficacy against *P. viticola* already at 10.0 mg/L, indicating that these molecules may play a key role in the anti‐oomycete activity of Russian sage extracts. Likewise, 400 mg L^−1^ carnosic acid, carnosol, and 12‐methoxycarnosic acid previously demonstrated inhibitory activities against *B. cinerea* and *Penicillium digitatum*.^55^ Moreover, 0.05 g L^−1^ carnosol can inhibit *Pyricularia oryzae*,^56^ and 0.05 g L^−1^ carnosic acid and its derivatives (11‐acetoxy carnosic acid and 11,12‐diacetoxy carnosic acid) can inhibit *A. alternata*, *B. cinerea*, and *F. oxysporum*,^26^ suggesting broad‐spectrum activity of these terpenoids. However, our compound annotation was limited to the most abundant features and reference standards tested, and low‐abundance compounds could also contribute to the anti‐oomycete activity of Russian sage extracts. Thus, further experiments are required to quantify bioactive compounds in alcoholic extracts, to investigate their mode of action, and to assess possible synergistic effects of compound mixtures under controlled and field conditions. Moreover, a large fraction of features of shoot extracts remained unannotated, indicating that further metabolomic and molecular network analyses are required to improve the annotation of Russian sage metabolites. A deeper understanding of compound accumulation in plant tissues is also required to optimize the extraction. For example, the accumulation of secondary metabolites in Russian sage varies during the vegetation season^31^ and it depends on environmental factors (e.g., light intensity, temperature, and water availability),^35^, ^57^ suggesting that growth conditions and harvesting time can be optimized to maximize bioactive compound yields.

## 5 CONCLUSION

Alcoholic extracts of Russian sage (*S. yangii*) decreased downy mildew severity on grapevine leaf disks, and treatments with 1.0%, 2.5%, and 5.0% shoot extract, 1.0% leaf extract, and 1.0% flower extract showed efficacy comparable to that obtained with copper‐based fungicides. Extract fractionation, leaf disk assays against *P. viticola*, and metabolomic analyses of active fractions identified putative terpenoids as the main components of active fractions. In particular, 7‐methylrosmanol, 12‐O‐methylcarnosic acid, carnosic acid, and carnosol resulted as bioactive compounds against *P. viticola*. Given the fast growth, adaptability, and low cost, Russian sage is a valuable plant resource for the development of sustainable plant protection products. Future research is required to identify additional bioactive compounds, to characterize the mode of action, and to optimize their extraction and formulation for field applications.

A.S. and O.G. prepared Russian sage extracts and carried out bioassays on leaf disks. A.S., P.R., and M.M. carried out the metabolomic analysis and metabolite annotations. A.S., P.R., and M.P. contributed to data interpretation. M.P. and P.R. conceived the study, designed the experiment, and coordinated all research activities. A.S., P.R., and M.P. wrote the manuscript. All the authors revised and approved the final manuscript.

## CONFLICT OF INTEREST

The authors declare no conflicts of interest.

## Supporting information


**Table S1.** Concentrations of treatments with alcoholic extracts of Russian sage.
**Table S2.** Annotated compounds detected by gas chromatography‐mass.
**Table S3.** Annotated compounds detected by ultra‐high‐pressure liquid.

**Figure S1.** The Compound Discoverer 3.2 workflow for untargeted metabolomics.
**Figure S2.** Comparison of the experimental mass spectra of annotated compounds.

## Data Availability

The data that supports the findings of this study are available in the supplementary material of this article.

## References

[1] Savary S , Willocquet L , Pethybridge SJ , Esker P , McRoberts N and Nelson A , The global burden of pathogens and pests on major food crops. Nat Ecol Evol 3:430–439, Nature Publishing Group (2019). 30718852 10.1038/s41559-018-0793-y

[2] Beckerman J , Palmer C , Tedford E and Ypema H , Fifty years of fungicide development, deployment, and future use. Phytopathology 113:694–706 (2023). 37137816 10.1094/PHYTO-10-22-0399-IA

[3] FAO , World Food and Agriculture – Statistical Yearbook 2023. FAO, Rome (2023), 10.4060/cc8166en.

[4] Fantke P , Friedrich R and Jolliet O , Health impact and damage cost assessment of pesticides in Europe. Environ Int 49:9–17 (2012). 22940502 10.1016/j.envint.2012.08.001

[5] Raveau R , Fontaine J and Lounès‐Hadj Sahraoui A , Essential oils as potential alternative biocontrol products against plant pathogens and weeds: a review. Foods 9:365 (2020). 32245234 10.3390/foods9030365 PMC7143296

[6] Paz C , Viscardi S , Iturra A , Marin V , Miranda F , Barra PJ *et al*., Antifungal effects of drimane sesquiterpenoids isolated from *Drimys winteri* against *Gaeumannomyces graminis* var. tritici, ed. by Druzhinina IS. Appl Environ Microbiol 86:e01834‐20 (2020). 33036992 10.1128/AEM.01834-20 PMC7688224

[7] Céspedes CL , Salazar JR , Ariza‐Castolo A , Yamaguchi L , Ávila JG , Aqueveque P *et al*., Biopesticides from plants: *Calceolaria integrifolia* s.l. Environ Res 132:391–406 (2014). 24893349 10.1016/j.envres.2014.04.003

[8] Wang H , Tian R , Chen Y , Li W , Wei S , Ji Z *et al*., In vivo and in vitro antifungal activities of five alkaloid compounds isolated from *Picrasma quassioides* (D. Don) Benn against plant pathogenic fungi. Pestic Biochem Physiol 188:105246 (2022). 36464333 10.1016/j.pestbp.2022.105246

[9] Koledenkova K , Esmaeel Q , Jacquard C , Nowak J , Clément C and Ait Barka E , *Plasmopara viticola* the causal agent of downy mildew of grapevine: from its taxonomy to disease management. Front Microbiol 13:889472 (2022). 35633680 10.3389/fmicb.2022.889472 PMC9130769

[10] Armijo G , Schlechter R , Agurto M , Muñoz D , Nuñez C and Arce‐Johnson P , Grapevine pathogenic microorganisms: understanding infection strategies and host response scenarios. Front Plant Sci 7:382 (2016). 27066032 10.3389/fpls.2016.00382 PMC4811896

[11] Dagostin S , Formolo T , Giovannini O , Pertot I and Schmitt A , *Salvia officinalis* extract can protect grapevine against *Plasmopara viticola* . Plant Dis 94:575–580 (2010). 30754462 10.1094/PDIS-94-5-0575

[12] Gessler C , Pertot I and Perazzolli M , *Plasmopara viticola*: a review of knowledge on downy mildew of grapevine and effective disease management. Phytopathol Mediterr 50:3–44 (2011).

[13] European Commission , Commission implementing regulation (EU) 2015/408 ‐ of 11 March 2015 ‐ on implementing Article 80(7) of Regulation (EC) No 1107/ 2009 of the European Parliament and of the Council concerning the placing of plant protection products on the market and establishing a list of candidates for substitution (2015).

[14] European Commission , Commission implementing regulation (EU) 2018/1981 ‐ of 13 December 2018 ‐ renewing the approval of the active substances copper compounds, as candidates for substitution, in accordance with Regulation (EC) No 1107 / 2009 of the European Parliament and of the Council concerning the placing of plant protection products on the market, and amending the Annex to Commission Implementing Regulation (EU) No 540 / 2011 (2018).

[15] Tamm L , Thuerig B , Apostolov S , Blogg H , Borgo E , Corneo PE *et al*., Use of copper‐based fungicides in organic agriculture in twelve European countries. Agronomy 12:673, Multidisciplinary Digital Publishing Institute (2022).

[16] Nadalini S and Puopolo G , Biological control of *Plasmopara viticola*: where are we now? in Biocontrol Agents for Improved Agriculture, ed. by Kumar A , Santoyo G and Singh J . Academic Press, Cambridge, MA, USA, pp. 67–100 (2024).

[17] Thuerig B , Ramseyer J , Hamburger M , Ludwig M , Oberhänsli T , Potterat O *et al*., Efficacy of a *Magnolia officinalis* bark extract against grapevine downy mildew and apple scab under controlled and field conditions. Crop Prot 114:97–105 (2018).

[18] Besrukow P , Will F , Berkelmann‐Löhnertz B , Stoll M and Schweiggert R , Efficacy of using grape cane extracts against *Plasmopara viticola* under field conditions and their impact on the composition of berries and musts of *Vitis vinifera* L. cv. Riesling. OENO One 58:4 (2024).

[19] Besrukow P , Will F , Dussling S , Berkelmann‐Löhnertz B and Schweiggert R , Additive and synergistic antifungal effects of copper and phenolic extracts from grape cane and apples. Pest Manag Sci 79:3334–3341 (2023). 37156732 10.1002/ps.7519

[20] Pébarthé‐Courrouilh A , Jaa A , Valls‐Fonayet J , Da Costa G , Palos‐Pinto A , Richard T *et al*., UV‐exposure decreases antimicrobial activities of a grapevine cane extract against *Plasmopara viticola* and *Botrytis cinerea* as a consequence of stilbene modifications—a kinetic study. Pest Manag Sci 80:6389–6399 (2024). 39172057 10.1002/ps.8367

[21] Pébarthé‐Courrouilh A , Jourdes M , Theil‐Bazingette M , Gancel A‐L , Teissedre P‐L , Valls‐Fonayet J *et al*., Antimicrobial properties of tannin extracts against the phytopathogenic oomycete *Plasmopara viticola* . OENO One 59:1–13 (2025).

[22] Thuerig B , James EE , Schärer H , Langat MK , Mulholland DA , Treutwein J *et al*., Reducing copper use in the environment: the use of larixol and larixyl acetate to treat downy mildew caused by *Plasmopara viticola* in viticulture. Pest Manag Sci 74:477–488 (2018). 28905481 10.1002/ps.4733

[23] Jakovljević M , Jokić S , Molnar M , Jašić M , Babić J , Jukić H *et al*., Bioactive profile of various *Salvia officinalis* L. preparations. Plants 8:55, Multidisciplinary Digital Publishing Institute (2019). 30845696 10.3390/plants8030055 PMC6473381

[24] Marchev A , Haas C , Schulz S , Georgiev V , Steingroewer J , Bley T *et al*., Sage in vitro cultures: a promising tool for the production of bioactive terpenes and phenolic substances. Biotechnol Lett 36:211–221 (2014). 24129950 10.1007/s10529-013-1350-z

[25] Han Y and Joo I , Antifungal effect of tanshinone from *Salvia miltiorrhiza* against disseminated candidiasis. YAKHAK HOEJI 57:119–124, The Pharmaceutical Society of Korea (2013).

[26] López‐Cabeza R , Rodríguez‐Sabina S , Reyes CP , Expósito DG , Giménez C , Jiménez IA *et al*., Bio‐guided isolation of aromatic abietane diterpenoids from *Salvia canariensis* as biopesticides in the control of phytopathogenic fungi. Pest Manag Sci 80:2199–2207 (2024). 38258969 10.1002/ps.7958

[27] Climati E , Mastrogiovanni F , Valeri M , Salvini L , Bonechi C , Mamadalieva NZ *et al*., Methyl carnosate, an antibacterial diterpene isolated from *Salvia officinalis* leaves. Nat Prod Commun 8:1934578X1300800402 (2013). 23738442

[28] Tada M , Okuno K , Chiba K , Ohnishi E and Yoshii T , Antiviral diterpenes from *Salvia officinalis* . Phytochemistry 35:539–541 (1994).

[29] Ivanov M , Kostić M , Stojković D and Soković M , Rosmarinic acid–modes of antimicrobial and antibiofilm activities of a common plant polyphenol. South Afr J Bot 146:521–527 (2022).

[30] Busato I , Bertaiola O , Tundo S , Guarnerio C , Lucchetta M , Sella L *et al*., A phytocomplex obtained from *Salvia officinalis* by cell culture technology effectively controls the grapevine downy mildew pathogen *Plasmopara viticola* . Plants 11:2675 (2022). 36297699 10.3390/plants11202675 PMC9606852

[31] Bielecka M , Stafiniak M , Pencakowski B , Ślusarczyk S , Jastrzębski JP , Paukszto Ł *et al*., Comparative transcriptomics of two *Salvia* subg. *Perovskia* species contribute towards molecular background of abietane‐type diterpenoid biosynthesis. Sci Rep 14:3046 (2024). 38321199 10.1038/s41598-024-53510-5 PMC10847172

[32] Bielecka M , Pencakowski B , Stafiniak M , Jakubowski K , Rahimmalek M , Gharibi S *et al*., Metabolomics and DNA‐based authentication of two traditional Asian medicinal and aromatic species of *Salvia* subg. Perovskia. Cells 10:112 (2021). 33435339 10.3390/cells10010112 PMC7826587

[33] Erdemgil FZ , Ilhan S , Korkmaz F , Kaplan C , Mercangöz A , Arfan M *et al*., Chemical composition and biological activity of the essential oil of Perovskia atriplicifolia. From Pakistan. Pharm Biol 45:324–331, Taylor & Francis (2007).

[34] Gostin IN and Popescu IE , Russian sage revealed: exploring biology, cultivation, and chemical dimensions of *Salvia yangii* . Agronomy 15:868, Multidisciplinary Digital Publishing Institute (2025).

[35] Kozłowska W , Matkowski A and Zielińska S , Light intensity and temperature effect on Salvia yangii (B. T. Drew) metabolic profile in vitro. Front Plant Sci 13:888509 (2022). 35646028 10.3389/fpls.2022.888509 PMC9136318

[36] Stafiniak M , Ślusarczyk S , Pencakowski B , Matkowski A , Rahimmalek M and Bielecka M , Seasonal variations of rosmarinic acid and its glucoside and expression of genes related to their biosynthesis in two medicinal and aromatic species of *salvia* subg. Perovskia. Biology 10:458 (2021). 34067387 10.3390/biology10060458 PMC8224735

[37] Afshari M , Rahimmalek M , Sabzalian MR , Bielecka M , Matkowski A and Talebi M , Changes in physiological, phytochemical traits and gene expression of two *Perovskia* species in response to water deficit. Sci Hortic 293:110747 (2022).

[38] Motyka S , Kusznierewicz B , Ekiert H , Korona‐Głowniak I and Szopa A , Comparative analysis of metabolic variations, antioxidant profiles and antimicrobial activity of *Salvia hispanica* (Chia) seed, sprout, leaf, flower, root and herb extracts. Molecules 28:2728, Multidisciplinary Digital Publishing Institute (2023). 36985699 10.3390/molecules28062728 PMC10056211

[39] Velickovic D , Randjelovic N , Ristic M , Velickovic A and Smelcerovic A , Chemical constituents and antimicrobial activity of the ethanol extracts obtained from the flower, leaf and stem of *Salvia officinalis* L. J Serbian Chem Soc 68:17–24 (2003).

[40] Perazzolli M , Moretto M , Fontana P , Ferrarini A , Velasco R , Moser C *et al*., Downy mildew resistance induced by *Trichoderma harzianum* T39 in susceptible grapevines partially mimics transcriptional changes of resistant genotypes. BMC Genomics 13:660 (2012). 23173562 10.1186/1471-2164-13-660 PMC3551682

[41] Avesani S , Lazazzara V , Robatscher P , Oberhuber M and Perazzolli M , Volatile linalool activates grapevine resistance against downy mildew with changes in the leaf metabolome. Curr Plant Biol 35–36:100298 (2023).

[42] Bolzonello A , Morbiato L , Tundo S , Sella L , Baccelli I , Echeverrigaray S *et al*., Peptide analogs of a *Trichoderma* peptaibol effectively control downy mildew in the vineyard. Plant Dis 107:2643–2652 (2023). 36724095 10.1094/PDIS-09-22-2064-RE

[43] EPPO , Guidelines for the efficacy evaluation of fungicides ‐ *Plasmopara viticola* . EPPO Bull 31:313–317 (2001).

[44] EPPO , Efficacy evaluation of plant protection products ‐ phytotoxicity assessment. EPPO Bull 44:265–273 (2014).

[45] Kim HW , Wang M , Leber CA , Nothias L‐F , Reher R , Kang KB *et al*., NPClassifier: a deep neural network‐based structural classification tool for natural products. J Nat Prod 84:2795–2807 (2021). 34662515 10.1021/acs.jnatprod.1c00399 PMC8631337

[46] Avesani S , Lazazzara V , Buti M , Oberhuber M , Robatscher P and Perazzolli M , Volatile 2‐Phenylethanol and β‐Cyclocitral trigger defense‐related transcriptional and metabolic changes in grapevine leaves against downy mildew. Physiol Plant 177:e70412 (2025). 40696500 10.1111/ppl.70412 PMC12284132

[47] Lazazzara V , Vicelli B , Bueschl C , Parich A , Pertot I , Schuhmacher R *et al*., *Trichoderma* spp. volatile organic compounds protect grapevine plants by activating defense‐related processes against downy mildew. Physiol Plant 172:1950–1965 (2021). 33783004 10.1111/ppl.13406 PMC8360165

[48] Widmer TL and Laurent N , Plant extracts containing caffeic acid and rosmarinic acid inhibit zoospore germination of *phytophthora* spp. pathogenic to *Theobroma cacao* . Eur J Plant Pathol 115:377–388 (2006).

[49] Montenegro I , Valenzuela Ormeño M , Seeger M , Besoain X , Godoy P , Werner E *et al*., Natural bactericidal effects of *Psoralea glandulosa* essential oil for the control of bacterial canker and speck in tomato. Agronomy 14:2615 (2024).

[50] Gong Y , Liu W , Huang X , Hao L , Li Y and Sun S , Antifungal activity and potential mechanism of N‐Butylphthalide alone and in combination with fluconazole against *Candida albicans* . Front Microbiol 10:1461, Frontiers Media Sa, Lausanne (2019). 31312187 10.3389/fmicb.2019.01461 PMC6614440

[51] Mongkol R and Chavasiri W , Antimicrobial, herbicidal and antifeedant activities of mansonone E from the heartwoods of *Mansonia gagei* Drumm. J Integr Agric 15:2795–2802 (2016).

[52] Giménez‐Martínez P , Cugnata N , Alonso‐Salces RM , Arredondo D , Antunez K , Castro RD *et al*., Short communication: natural molecules for the control of *Paenibacillus larvae*, causal agent of American foulbrood in honey bees (*Apis mellifera* L.), *Span* . J Agric Res 17:e05SC01 (2019).

[53] Dekić MS , Gusinac AM , Jeremić SR , Jakovljević VD , Plojović SA , Jovanović D *et al*., Abietane diterpenoid salvipisone: structure elucidation, conformational analysis, and antimicrobial activity. J Mol Struct 1321:139807 (2025).

[54] Krishna Kumari G and Zaluzanin D , A fungistatic sesquiterpene from *Vernonia arborea* . Fitoterapia 74:479–482 (2003). 12837366 10.1016/s0367-326x(03)00054-6

[55] Exarchou V , Kanetis L , Charalambous Z , Apers S , Pieters L , Gekas V *et al*., HPLC‐SPE‐NMR characterization of major metabolites in *Salvia fruticosa* mill. Extract with antifungal potential: relevance of carnosic acid, carnosol, and hispidulin. J Agric Food Chem 63:457–463, American Chemical Society (2015). 25537192 10.1021/jf5050734

[56] Ramírez J , Gilardoni G , Ramón E , Tosi S , Picco AM , Bicchi C *et al*., Phytochemical study of the Ecuadorian species *Lepechinia mutica* (Benth.) Epling and high antifungal activity of carnosol against *Pyricularia oryzae* . Pharmaceuticals 11:33(2018). 29671794 10.3390/ph11020033 PMC6027405

[57] Khodadadi F , Ahmadi FS , Talebi M , Moshtaghi N , Matkowski A , Szumny A *et al*., Essential oil composition, physiological and morphological variation in *Salvia abrotanoides* and *S. Yangii* under drought stress and chitosan treatments. Ind Crops Prod 187:115429 (2022).

