# Supplementary material for: Alcoholic extracts of Russian sage ( Salvia yangii ) contain bioactive terpenoids with inhibitory activity against grapevine downy mildew ( Plasmopara viticola )
Source: Pest Manag Sci. 2026 May 1;82(9):8159–70. doi: 10.1002/ps.70869 (PMC13453268; doi:10.1002/ps.70869)
Supplement: Supplementary file 1 [file PS-82-8159-s001.pdf]

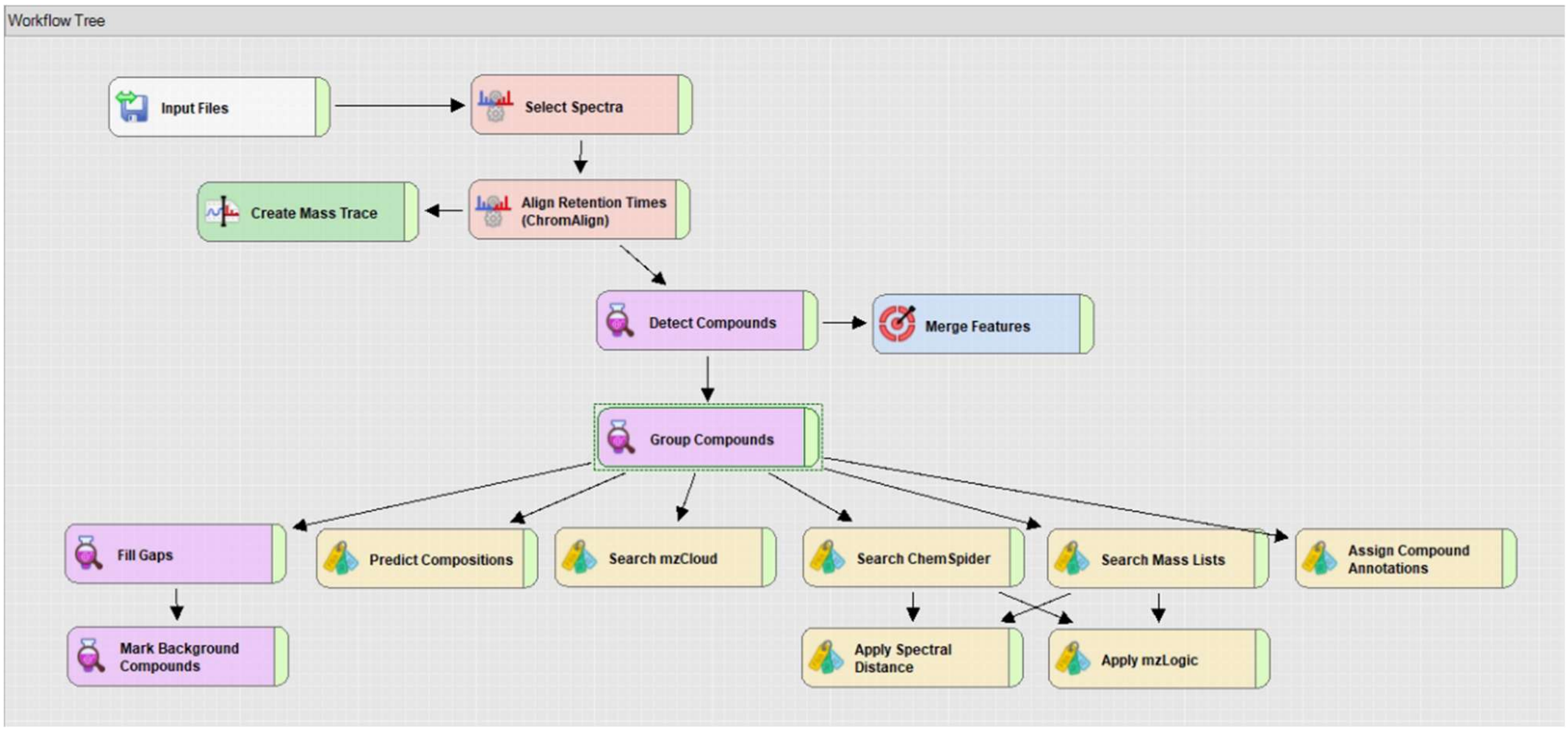

Supplementary Figure S1. The Compound Discoverer 3.2 workflow for untargeted metabolomics. Shoot extract fractions of Russian sage were analyzed using ultra-high-pressure liquid chromatography-high resolution mass spectrometry (UHPLC-HRMS). Full scan MS data and ddMS^2 fragmentation data were processed using an untargeted metabolomics workflow of Compound Discoverer. The nodular workflow involved spectrum selection (Select Spectra), chromatographic alignment (Align Retention Times), mass chromatogram extraction (Create Mass Trace), feature detection (Detect Compounds, Merge features, Group Compounds, and Fill Gaps), peak area normalization (Mark Background Compounds), chemical composition prediction (Predict Compositions), and chemical annotation of each feature (Search mzCloud, Search ChemSpider, Search Mass Lists, Apply mzLogic, Apply Spectral Distance, and Assign Compound Annotations).

To ensure data quality, samples were randomized during the injection sequence, and blank [corresponding to a methanol:water (50:50; v/v) solution] injections were included every ten samples to remove background signals. Features were detected (Detect Compounds) using m/z range 90-1,350, mass tolerance of 5 ppm, minimum peak intensity of 10,000, and signal-to-noise ratio of 1.5. Data normalization was carried out according to peak area. Features were merged across samples with a mass tolerance of 5 ppm and retention time tolerance of 0.1 min, and grouped with a peak rating threshold ≥5 in at least one file. Features of interest were selected by imposing an abundance ratio greater than two in pairwise comparisons between active and non-active fractions against *P. viticola*, while ensuring low variability among active fractions with an abundance ratio lower than two in the pairwise comparisons among active fractions.

A

7-methylrosmanol

Russian sage extract measured retention time: 11.55 min
Reference standard measured retention time: 11.55 min

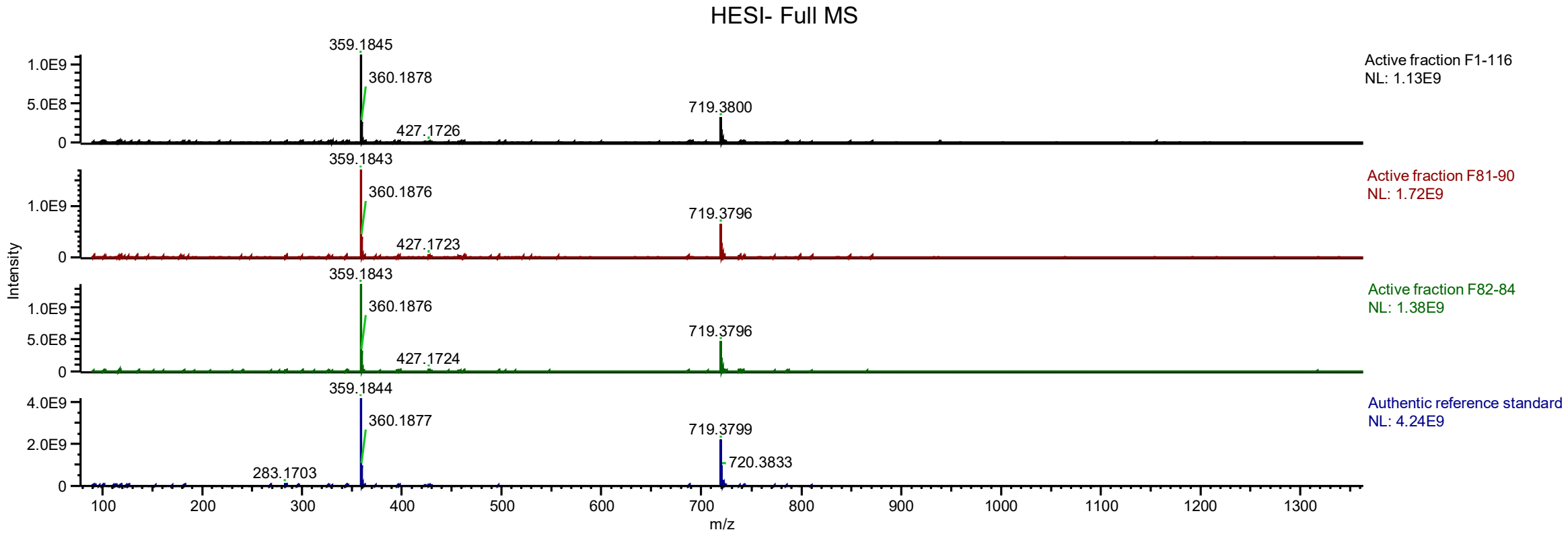

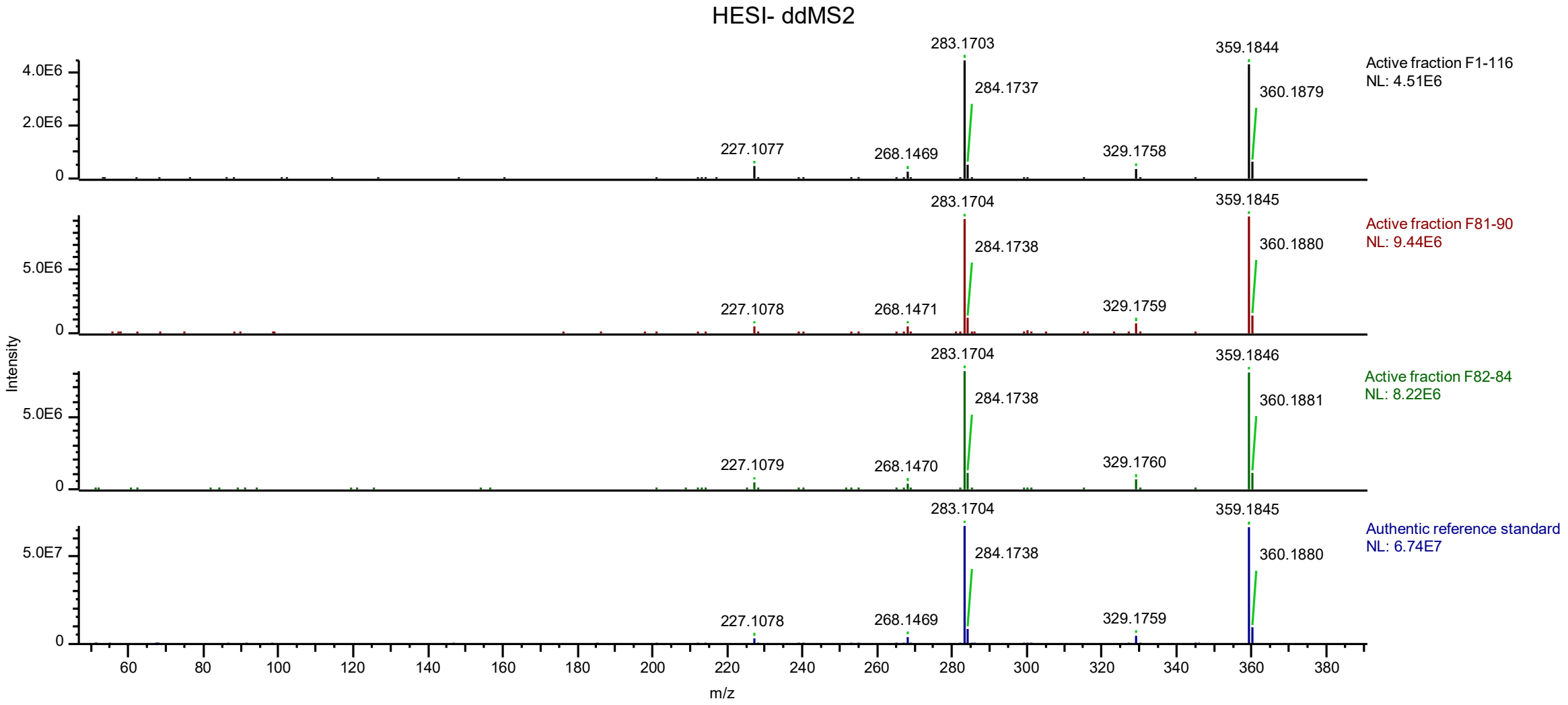

B

12-O-methylcarnosic acid

Russian sage extract measured retention time: 11.47 min
Reference standard measured retention time: 11.47 min

HESI- Full MS

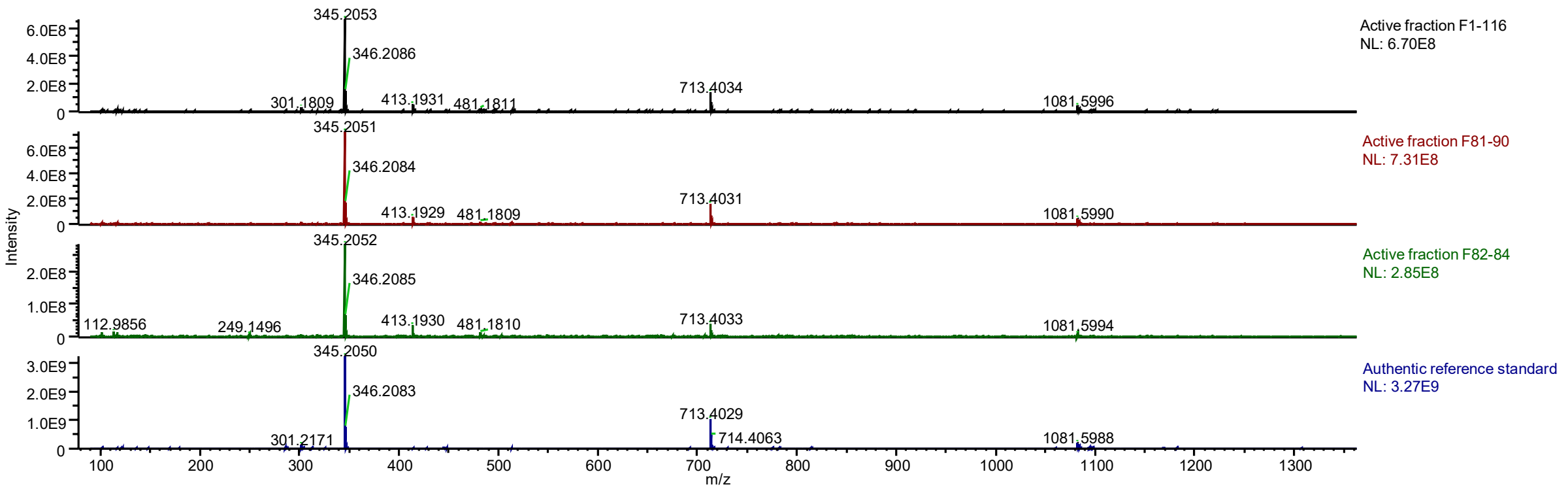

HESI- ddMS2

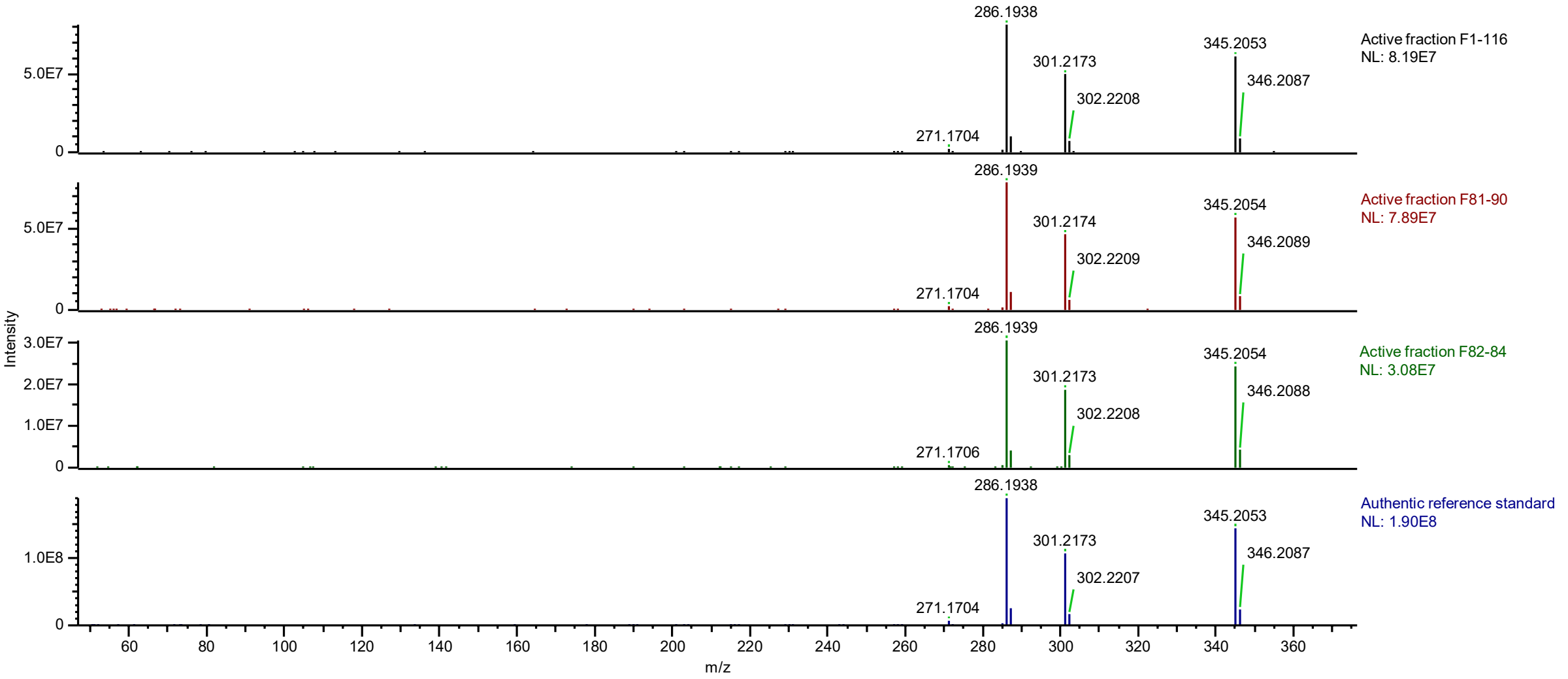

C

carnosic acid

Russian sage extract measured retention time: 13.09 min
Reference standard measured retention time: 13.09 min

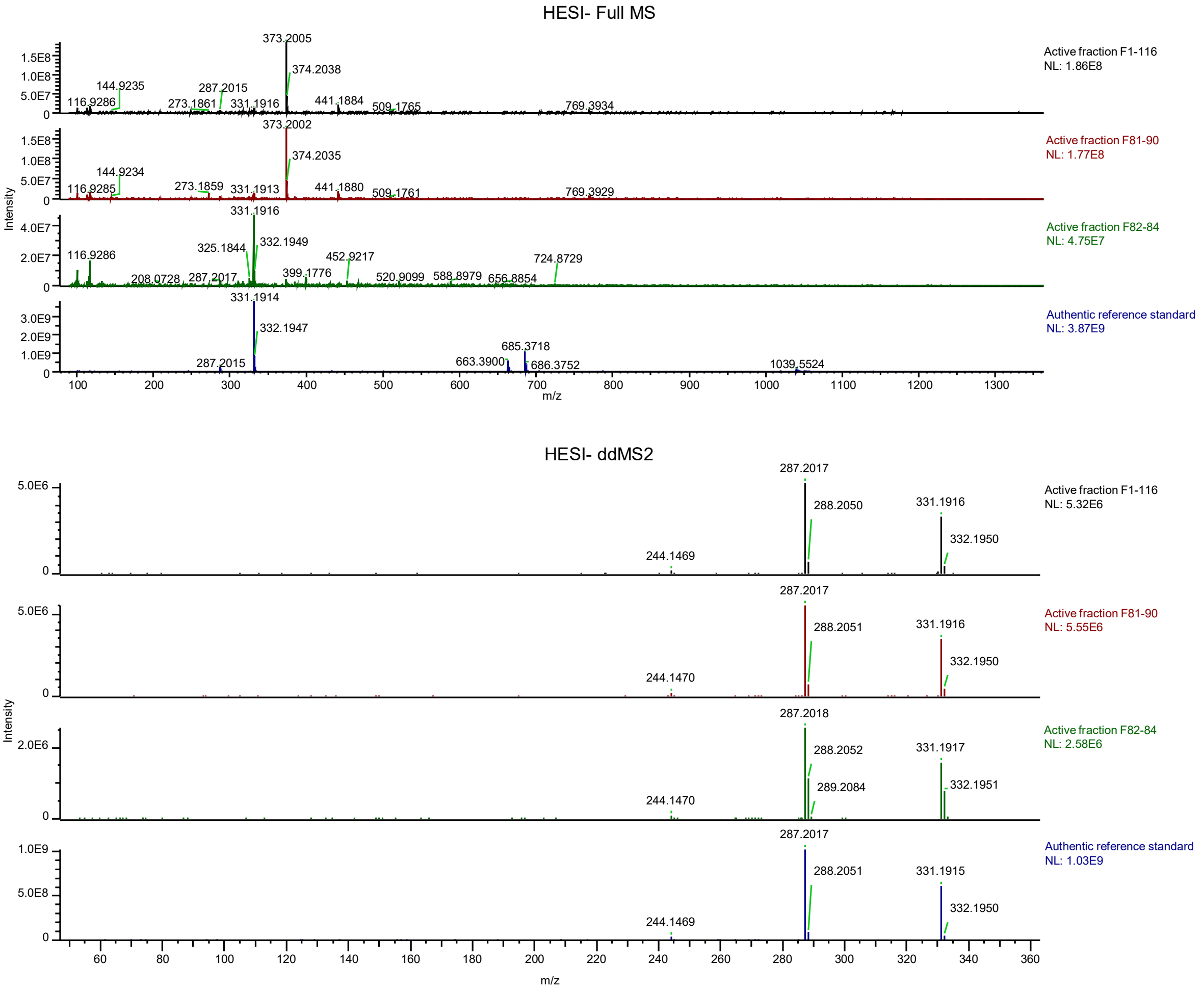

D **carnosol**

Russian sage extract measured retention time: 11.33 min
Reference standard measured retention time: 11.33 min

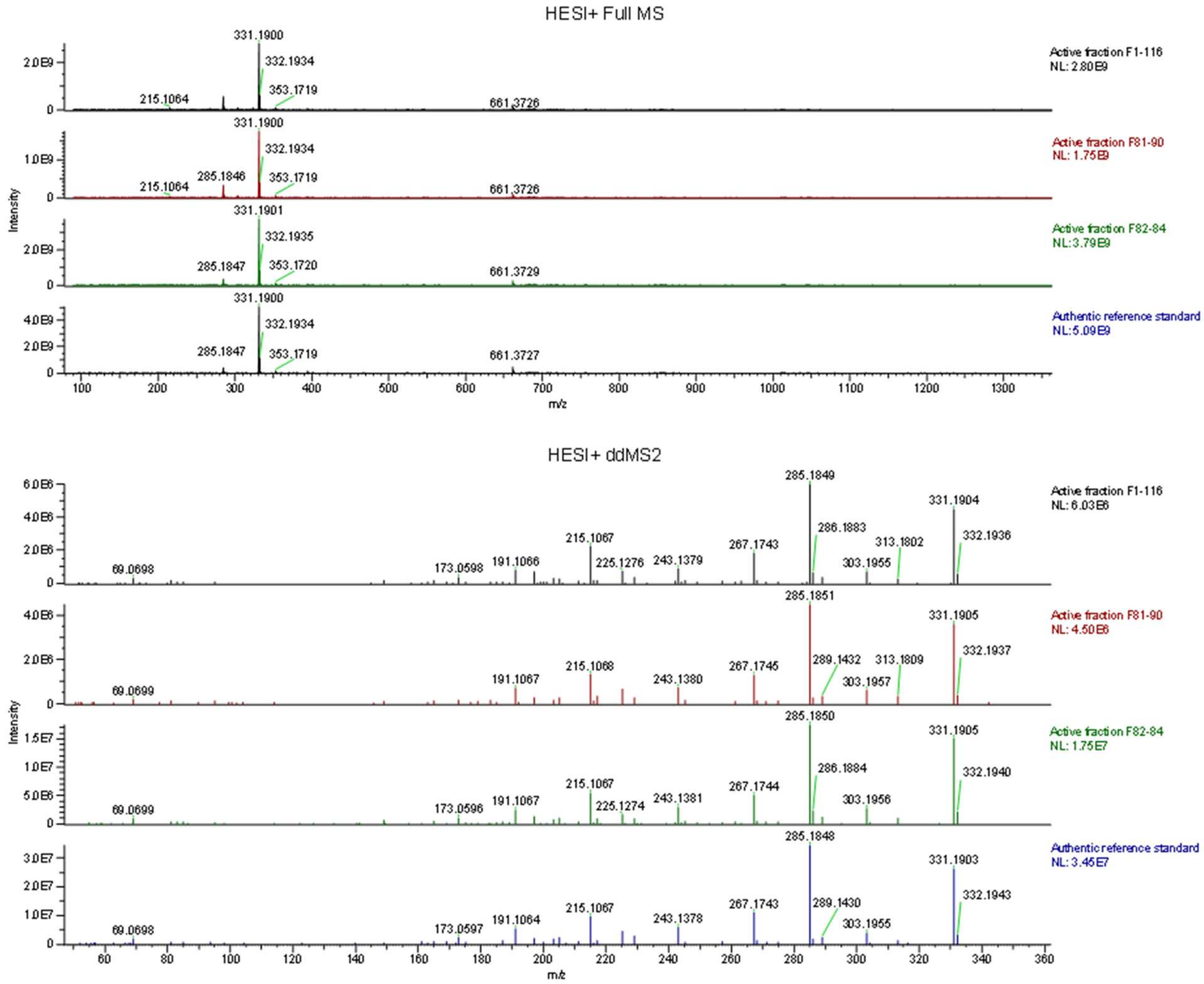

E

carnosol

Russian sage extract measured retention time: 11.32 min
Reference standard measured retention time: 11.32 min

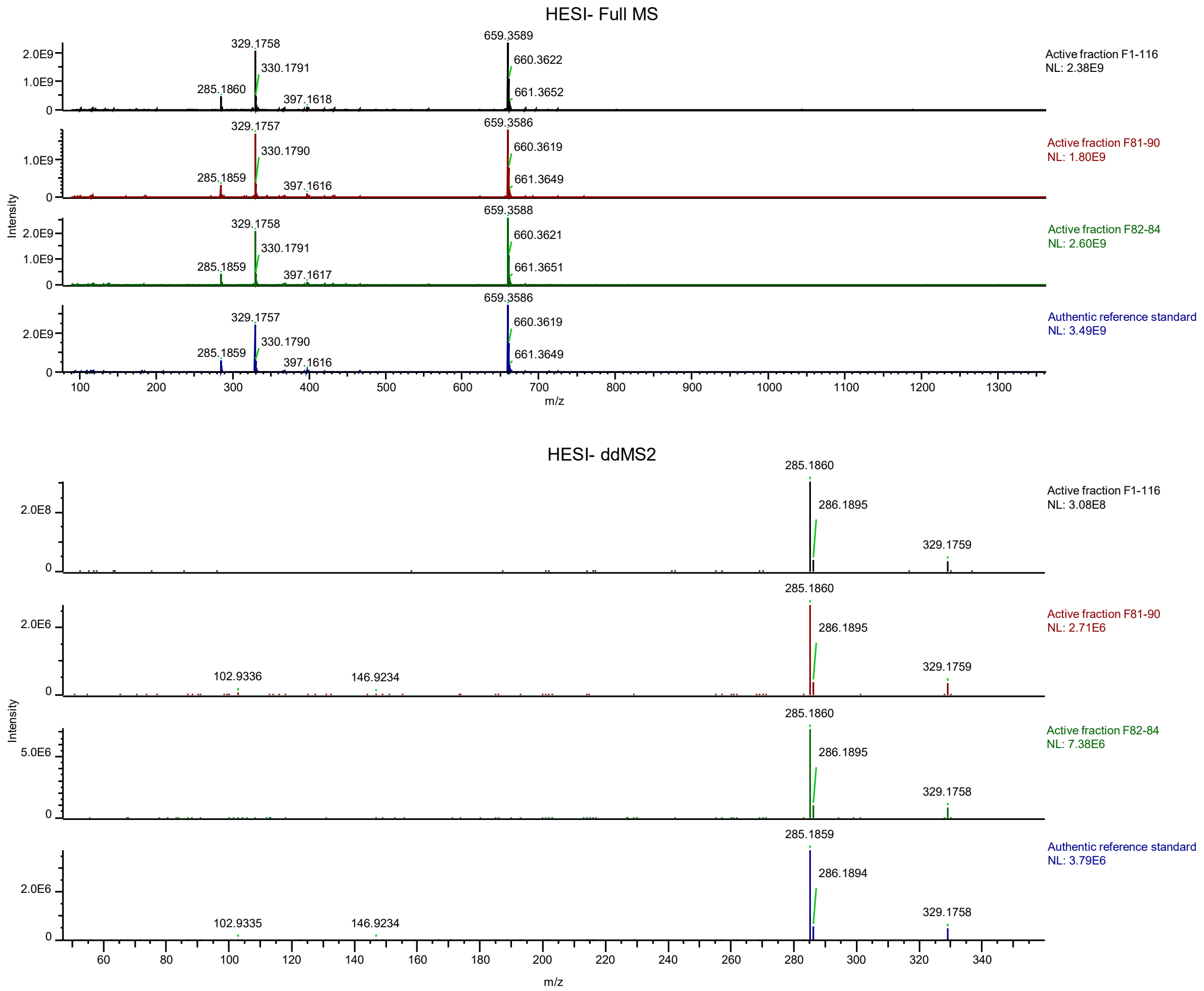

F

maslinic acid

Russian sage extract measured retention time: 12.41 min
Reference standard measured retention time: 12.41 min

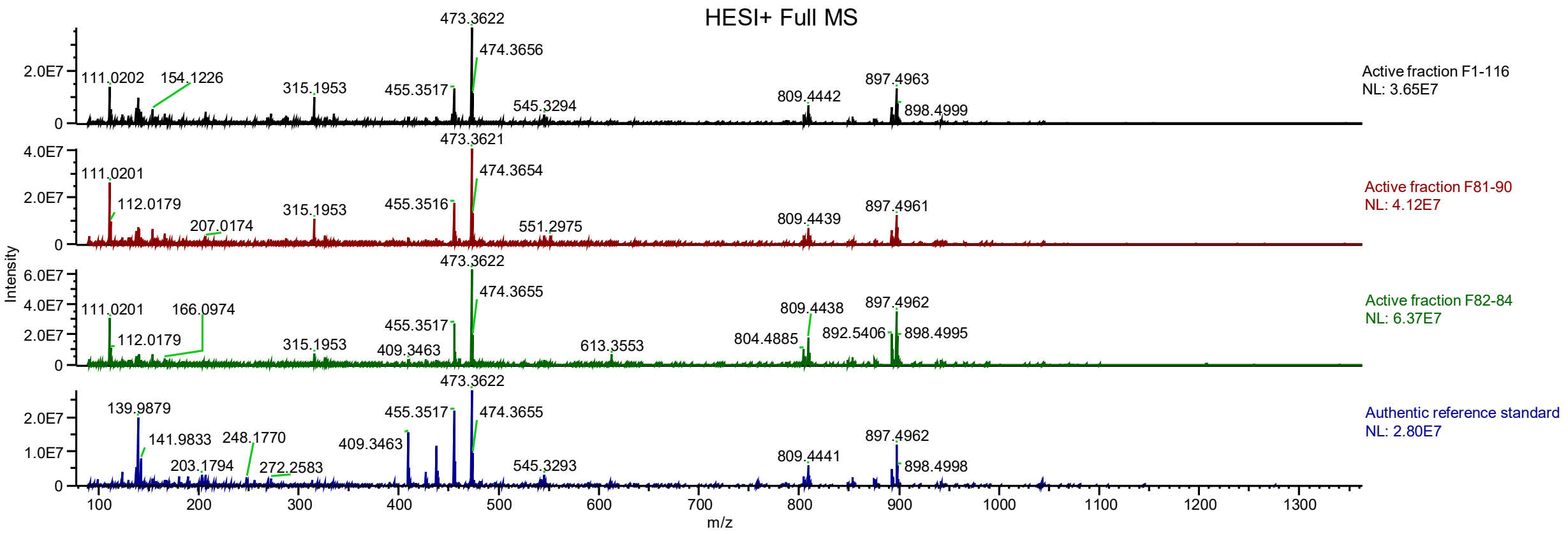

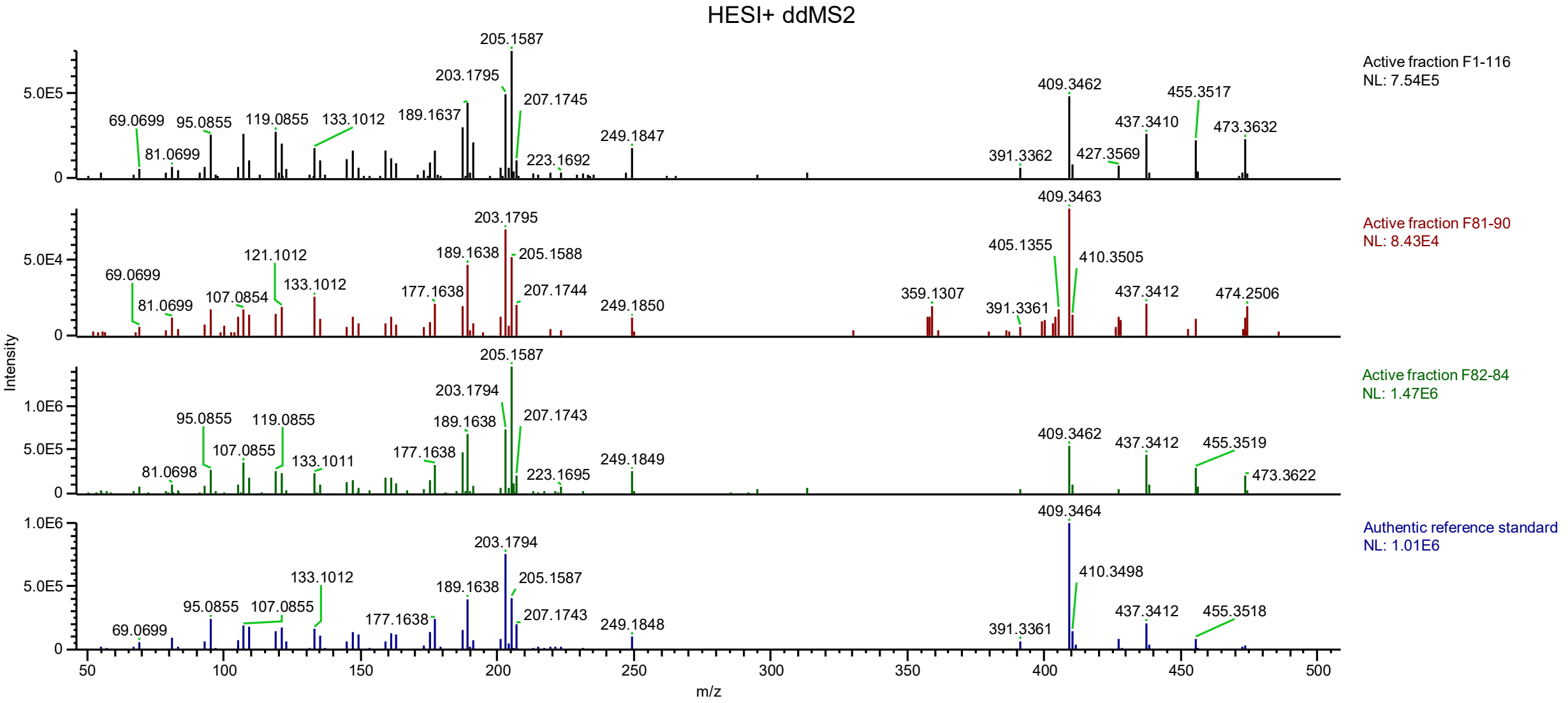

G **maslinic acid**

Russian sage extract measured retention time: 12.41 min
Reference standard measured retention time: 12.41 min

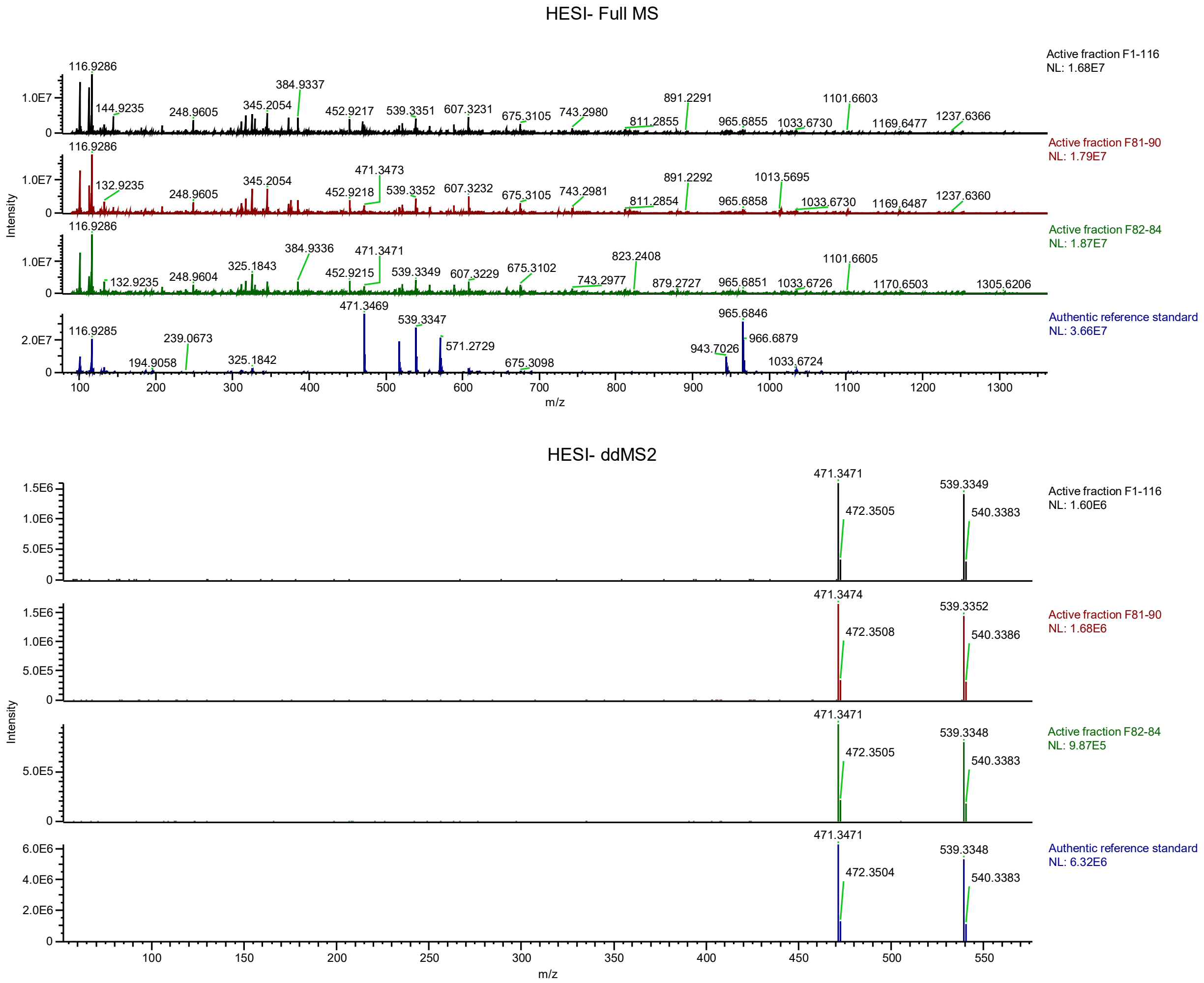

Supplementary Figure S2. Comparison of the experimental mass spectra of annotated compounds found in alcoholic extracts of Russian sage (active fractions F1-116, F81-90 and F82-84) with the experimental mass spectra of the corresponding reference standard, such as 7-methylrosmanol (A), 12-O-methylcarnosic acid (B), carnosic acid (C), carnosol (D and E in positive and negative HESI mode, respectively), and maslinic acid (F and G in positive and negative HESI mode, respectively). The comparison of full scan mass (MS) spectra and data-dependent tandem mass spectrometry ($ddMS^2$) fragmentation spectra, retention time, heated electrospray ionization (HESI) mode, and normalized intensity levels (NL) is reported for each compound.

Supplementary Table S1. Concentrations of treatments with alcoholic extracts of Russian sage. Treatment abbreviation (column A), treatment description (column B), and application concentration (column C) are reported for each treatment with alcoholic extracts of Russian sage on grapevine leaf disks.

Alcoholic extracts of Russian sage were obtained from 20 g of dried powder of shoots [57% (w/w) leaves, 14% (w/w) flowers, and 29% (w/w) stems], flowers, leaves, or stems in 400 mL of 99.8% (v/v) ethanol using a Soxhlet extractor. Each dried extract was resuspended in 20 mL (shoot and stem extracts) or 40 mL (flower and leaf extracts) of 99.8% (v/v) ethanol to obtain the acholic extract (column D). The shoot extract was fractionated using a preparative liquid chromatography with a C18 column, and fractions obtained from two independent runs were combined as pools of ten fractions (10-fraction pools; from F1-10 to F91-116), three fractions (3-fraction pools; from F55-57 to F88-90), or 116 fractions (reconstructed shoot extract, F1-116). Each pool of shoot extract fractions was freeze-dried and resuspended in 0.5 mL 99.8% (v/v) ethanol. Alcoholic extracts (column E) were mixed with ethanol (column F), DMSO (column G), and water (column H) to obtain 10 mL of treatment solutions, containing 10% ethanol and 0.5% DMSO. Different concentrations of shoot extracts [0.5, 1, 2.5, or 5% (w/v)] were tested. Leaf, flower, and stem extracts were used at 1% (w/v), corresponding to the minimum concentration of shoot extract with efficacy comparable to that of the reference fungicide. Each pool of shoot extract fractions was applied at 5% (w/v), corresponding to the maximum efficacy obtained with shoot extracts. Copper and a solution of 10% ethanol and 0.5% DMSO in water (Control) were used in each experiment as reference fungicide and control, respectively.

Concentrations of alcoholic extracts and shoot extract fractions were expressed as the weight (g) of dry biomass of shoot, flower, leaf, or stem samples resuspended in 100 mL of treatment solution (10.0% ethanol and 0.5% DMSO in water) after extraction, drying, resuspension, and fractionation, according to the calculations reported in columns E-H.

Supplementary Table S2. Annotated compounds detected by gas chromatography-mass spectrometry (GC-MS) analysis.

Selected fractions of Russian sage were analyzed by GC-MS, and full scan MS data were processed using the GC-MS solution software to annotate features detected in all active fractions (F1-116, F81-90, and F82-84), but not detected in non-active fractions (F1-10 and F91-116), according to visual observation of chromatograms.

Compound annotation was carried out by comparing full scan MS data to reference spectra of NIST 2017, imposing a minimum spectral similarity of 75% and annotated compounds were classified into putative chemical classes according to the classification obtained with the NP classifier web-based

application (https://npclassifier.ucsd.edu/). The putative chemical name (column B), putative chemical formula (column C), mean retention time (column D), spectral similarity (column E), database ID (column F), chemical class (columns G and H), and organism where the compound was previously found (column I) are reported for each annotated compound.

Supplementary Table S3. Annotated compounds detected by ultra-high-pressure liquid chromatography-high resolution mass spectrometry (UHPLC-HRMS) analysis.
Selected fractions of Russian sage were analyzed by UHPLC-HRMS, and full scan MS and $ddMS^2$ fragmentation data were processed using an untargeted metabolomics workflow of Compound Discoverer software. Features of interest, specified by mean retention time (column C) and mean mass-to-charge ratio (*m/z*; column D), were selected imposing an abundance ratio greater than two in the pairwise comparisons between active and non-active fractions against *P. viticola*, and an abundance ratio lower than two in the pairwise comparisons among active fractions. The abundance (peak area expressed as counts per second) of each feature is reported for active (F1-116, F81-90, and F82-84) and non-active (F1-10 and F91-116) fractions of Russian sage (columns O-S).
Reference ion (column E) and fragmentation information (column F) indicate when $ddMS^2$ fragmentation spectra were obtained for the preferred ion (e.g., $[M+H]^{+1}$ or $[M-H]^{-1}$) or another ion (e.g., $[M+2H]^{+2}$, $[M+FA-H]^{-1}$, $[M+H-H_2O]^{+1}$, $[M-H-H_2O]^{-1}$, or $[M+Na]^{+1}$).
Molecular weight (column G), predicted chemical formula (column H), delta mass error between the measured molecular weight and molecular weight calculated from the predicted chemical formula (column I) are reported for the 100 most abundant features (column B) detected in positive (HESI +) and negative (HESI -) HESI mode (column A).

Molecular weight and predicted chemical formula of the most abundant features were searched in PubChem (https://pubchem.ncbi.nlm.nih.gov/), LOTUS (https://lotus.naturalproducts.net/), PlantaeDB (https://plantaedb.com/), and KNApSAcK (https://www.knapsackfamily.com/KNApSAcK/) databases to retrieve putative chemical name (column J). Database reference spectra and in silico fragmentation spectra obtained with Compound Discoverer software were visually compared with the experimental full scan MS spectra and $ddMS^2$ fragmentation spectra to confirm the putative chemical name of the most abundant features (annotated compounds). Annotated compounds were classified into 13 putative chemical classes (column K) and detailed chemical classes (column L), according to classification obtained with the NP classifier web-based application (https://npclassifier.ucsd.edu/).
The organism where the compound was previously found (column M) and database ID (column N) are reported for each annotated compound. Compound annotations validated by reference standards are reported in bold.
